# Effect of Arctic Service Conditions on the Mechanical Properties and Damage Behavior of Glass Fiber and Carbon/Glass Hybrid-Reinforced Vinyl Ester Composites for Marine Applications

**DOI:** 10.3390/polym18162002

**Published:** 2026-08-17

**Authors:** Lijun Wang, Yueming Zhou, Weiping He, Xin Fu, Zhiyong Zhao, Xingyue Zhen, Bin Yang, Jihui Wang, Aiqing Ni

**Affiliations:** 1 School of Materials Science and Engineering, Wuhan University of Technology, Wuhan 430070, China; 2 Wuhan Second Ship Design and Research Institute, Wuhan 430061, China; 3 State Key Laboratory of Advanced Technology for Materials Synthesis and Processing, Wuhan University of Technology, 122 Luoshi Road, Wuhan 430070, China

**Keywords:** glass fiber reinforced composites, carbon/glass hybrid composites, vinyl ester resin, freeze–thaw cycling, low-temperature exposure, mechanical properties, glass transition temperature, moisture preconditioning

## Abstract

Glass fiber-reinforced polymer (GFRP) and carbon/glass hybrid fiber-reinforced polymer (HFRP) laminates with two vinyl ester resin systems were investigated to evaluate their early environmental response, residual mechanical performance, and damage behavior after moisture-assisted low-temperature exposure and freeze–thaw cycling (FTC). After 150 days of moisture preconditioning, the conditioned specimens were exposed to −50 °C or subjected to FTC between −50 °C and 22 °C. Tensile, compressive, flexural, in-plane shear, interlaminar shear, and compression-after-impact (CAI) tests were conducted. Fourier transform infrared spectroscopy (FTIR), dynamic mechanical analysis (DMA), and scanning electron microscopy (SEM) were used to examine chemical structure, thermomechanical response, and damage morphology. FTIR spectra showed no obvious changes in the characteristic absorption bands of the vinyl ester matrix. DMA showed condition-dependent changes in thermomechanical behavior, with the largest decrease in glass transition temperature reaching 5.5 °C after Condition 3. Tensile and in-plane shear properties were largely retained, whereas compressive, flexural, interlaminar shear, and CAI properties were more sensitive; the largest CAI strength loss was 19.1%. Hybrid stacking affected the property retention and damage tolerance of the laminates under the designed FTC condition. SEM observations identified interfacial debonding, matrix microcracking, and interlaminar crack growth as the main damage features.

## 1. Introduction

In recent years, fiber-reinforced polymer (FRP) composites have been increasingly used in marine structures, such as ships, offshore platforms, offshore wind turbine components, underwater vehicles, and marine civil infrastructure, owing to their low density, high specific strength, corrosion resistance, and design flexibility. Compared with conventional metallic materials, glass fiber reinforced polymer (GFRP) composites and carbon fiber reinforced polymer (CFRP) composites can reduce structural weight, improve resistance to seawater corrosion, lower maintenance requirements, and enable integrated fabrication of complex components, making them promising candidates for lightweight structural applications in polar marine equipment [1,2,3,4,5].

With the expansion of marine engineering applications from conventional offshore regions to high-latitude and polar seas, the service reliability and long-term durability of FRP composites in low-temperature marine environments have attracted increasing attention. Polar marine environments are generally characterized by the combined action of seawater immersion, temperature fluctuations, low-temperature exposure, repeated freeze–thaw cycling (FTC), and cyclic structural loading. These factors may induce matrix cracking, interfacial weakening, and interlaminar damage, thereby affecting the mechanical performance and service safety of FRP composites [6]. Previous studies have shown that moisture absorption is not merely associated with mass uptake, but can also alter the microstructural state of the resin matrix and fiber–matrix interface through water diffusion [7,8]. Moisture ingress may induce matrix plasticization, hygroscopic swelling, molecular relaxation, and possible local chemical deterioration [9,10], thereby weakening fiber–matrix interface bonding and reducing the efficiency of load transfer across the interface [11,12,13,14,15]. Similar hydrothermal aging studies on fiber-reinforced polymer composite pipes also showed that water absorption can be closely related to the degradation of mechanical properties, further highlighting the importance of considering moisture history in durability evaluation [16]. Under freezing conditions, moisture retained in the resin matrix, voids, and interfacial defects may induce local internal stresses and volumetric expansion, further promoting the propagation of pre-existing microcracks and interfacial defects [17]. These damage processes are particularly relevant to matrix- and interface-dominated properties, such as flexural strength, compressive strength, interlaminar shear strength and residual compression-after-impact (CAI) strength [4,15,18,19].

Several studies have investigated the durability of GFRP composites subjected to FTC, with existing studies mainly focusing on GFRP plates and profiles fabricated by pultrusion, hand lay-up or vacuum infusion. Hasan et al. [18] investigated the freeze–thaw durability of vacuum-infused GFRP laminates after distilled-water preconditioning. Their results showed that freeze–thaw exposure caused slight-to-moderate reductions in the glass transition temperature ($T_{g}$) and mechanical properties, with flexural and compressive strengths showing more pronounced reductions. Previous studies have suggested that freeze–thaw induced degradation is mainly associated with matrix plasticization, thermal expansion mismatch between fibers and resin, volumetric expansion of freezing water and fiber/resin interfacial damage. Compared with thermal cycling, FTC in the presence of absorbed moisture is more likely to accelerate internal damage accumulation and cause mechanical property deterioration [18,20]. In addition, comparative studies on CFRP and GFRP composites have shown that GFRP composites may suffer from tensile property degradation and fiber/resin interfacial damage after FTC. However, the extent of degradation is closely related to the resin system, fiber architecture, manufacturing process, moisture condition and freeze–thaw protocol [13]. Shakiba et al. [12] reported that GFRP stirrups exhibited strength degradation after freeze–thaw exposure, and that seawater FTC caused more pronounced deterioration than tap-water FTC. Although this study focused on GFRP stirrups rather than laminates, the results indicate that the surrounding aqueous medium can influence FTC-induced degradation [12,21].

Monolithic CFRP composites generally exhibit high stiffness and specific strength, but their high material cost limits their broader use in structural applications. In contrast, monolithic GFRP composites are more cost-effective and damage-tolerant, but their load-bearing efficiency and lightweight potential are relatively limited. Carbon/glass hybridization provides an effective strategy for developing hybrid fiber reinforced polymer (HFRP) composites with a balanced combination of stiffness, load-bearing capacity, damage tolerance and cost-effectiveness [22], thereby showing considerable potential for marine decks, hull structures, yacht components and other lightweight marine engineering applications [23,24]. Zhang et al. [25] prepared glass/carbon woven hybrid laminates and evaluated their tensile, compressive and flexural properties, showing that selective incorporation of carbon fibers into GFRP laminates can achieve a low-cost and high-strength lightweight structure. Monjon et al. [26] compared hybrid laminates with monolithic CFRP, GFRP and Kevlar/epoxy laminates through bending and interlaminar shear tests, demonstrating that hybridization provides a more balanced combination of stiffness, strength and deformation capacity than single-fiber laminates. Previous studies on the moisture absorption behavior of HFRP composites have indicated that hybrid architecture is closely related to water diffusion behavior and residual mechanical performance after moisture exposure. Mohanty et al. [27] reported that the incorporation of carbon fibers reduced the moisture sensitivity of GFRP composites in hot-water environments, while moisture ingress degraded the load-bearing capacity of the resin matrix and fiber/resin interface. Jesthi and Nayak [28] showed that carbon/glass hybridization reduced seawater uptake and diffusion in HFRP laminates, although moisture ingress still induced matrix swelling, interfacial debonding and reductions in residual tensile, flexural and impact properties. Zhou et al. [29] reported that moisture preconditioning and FTC caused mechanical property degradation in both GFRP and HFRP composites, whereas an appropriate combination of carbon fiber type and hybrid stacking configuration improved the property retention of HFRP composites under freeze–thaw exposure. Although these studies have improved the understanding of FRP durability under seawater immersion and freeze–thaw environments, the mechanical property evolution and degradation mechanisms of carbon/glass HFRP composites under low-temperature freeze–thaw conditions remain insufficiently understood. To provide a clearer comparison of the existing studies, representative investigations on GFRP, CFRP and carbon/glass HFRP composites under low-temperature or FTC-related conditions are summarized in Table 1. These studies indicate that the environmental response of FRP composites depends strongly on the fiber type, resin matrix, hybrid architecture, moisture condition and testing method.

Building on the material systems reported by Zhou et al. [29], the present study further considers a different environmental history and a damage-tolerance-related response. Instead of using accelerated moisture conditioning at elevated temperature followed by multiple FTC stages, the specimens in this work were subjected to 150 days of immersion at 22 °C before subsequent −50 °C exposure or moisture-assisted FTC. CAI testing was also introduced to evaluate the residual compressive load-bearing capacity after impact damage, which was not addressed in the previous study. Therefore, this work focuses on how the resin system and carbon/glass hybrid architecture affect the early residual mechanical response, damage tolerance, and damage evolution of GFRP and HFRP laminates under the designed environmental histories. All laminates were fabricated using the vacuum-assisted resin infusion (VARI) process, and their residual properties were evaluated through tensile, compressive, flexural, in-plane shear, interlaminar shear, CAI, DMA, FTIR, and SEM analyses.

## 2. Materials and Methods

### 2.1. Materials

Two vinyl ester resin systems were used in this study: a commercial 430LV vinyl ester resin, designated as VE-1, supplied by Nanjing Jinling DSM Co., Ltd. (Nanjing, China), and a modified vinyl ester resin with an optimized formulation based on 430LV, designated as VE-2. The detailed formulation of VE-2 is proprietary to the supplier and is therefore not disclosed in this study. Aksu Butanox M50 (Nouryon, Tianjin, China), mainly composed of methyl ethyl ketone peroxide (MEKP), was used as the initiator, and E4 (Changzhou Tianma Group Co., Ltd., Changzhou, China) was used as the accelerator in the curing system. For both resin systems, the resin, initiator, and accelerator were mixed at a mass ratio of 100:2:0.2. The basic properties of the cured resins are summarized in Table 2.

The reinforcing materials included one glass fiber fabric and two carbon fiber fabrics. The glass fiber fabric was a high-strength E-glass satin weave fabric, designated as GF, with the model number SW220C-100b, and was developed by the Nanjing Glass Fiber Research and Design Institute. Two types of carbon fiber fabrics were employed: CF-1 was a T300-grade twill-woven fabric, whereas CF-2 was a T700-grade non-crimp fabric (NCF) with carbon fiber bundles arranged in the 0° and 90° directions. The basic properties of the fibers are summarized in Table 3.

### 2.2. Specimen Preparation

**Figure 1:**
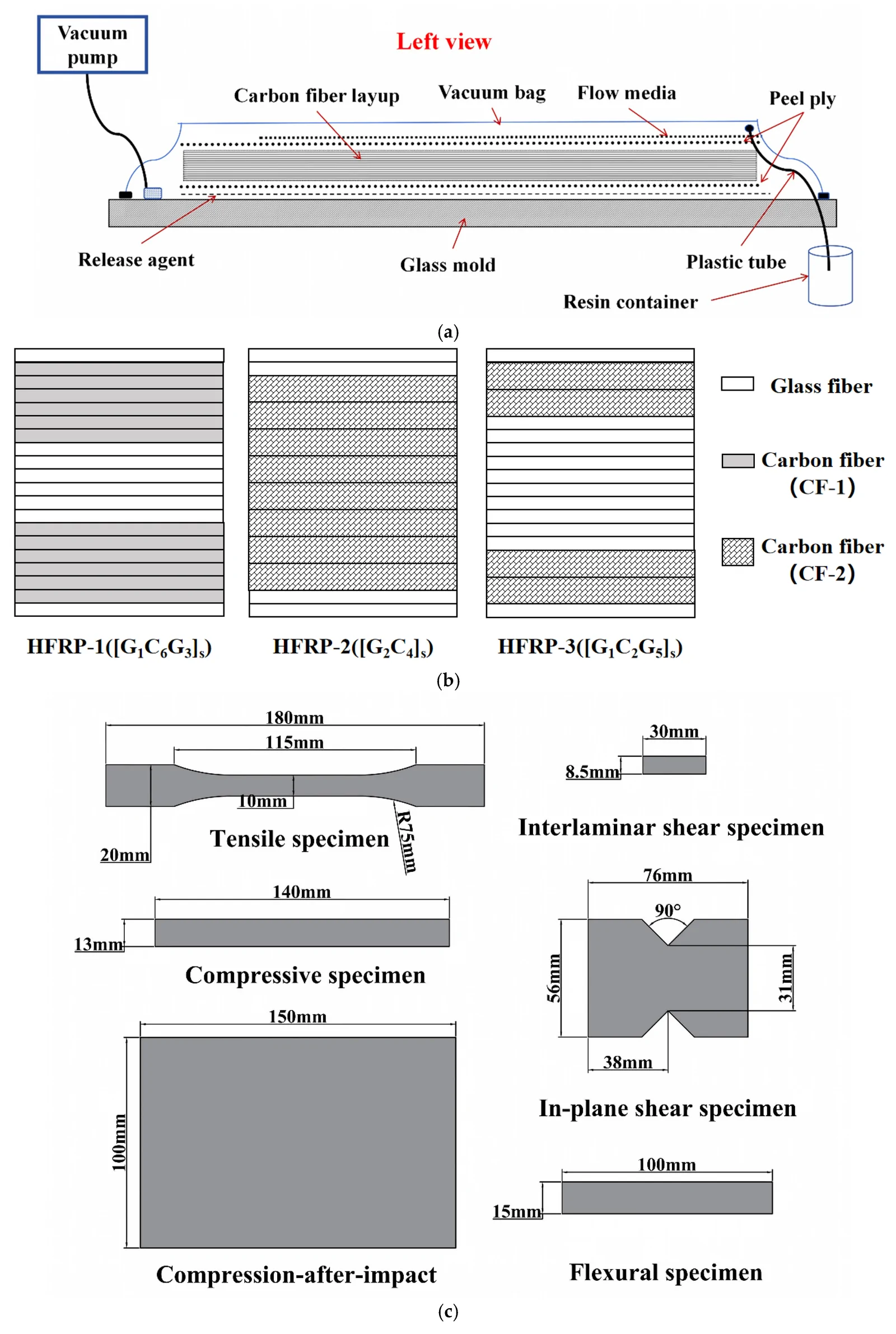
(**a**) Schematic of the VARI fabrication process, (**b**) schematic indicating the lay-up configurations of HFRP composites, and (**c**) schematic of standard mechanical test specimen dimensions.

All laminates were fabricated using the vacuum-assisted resin infusion (VARI) process, as illustrated in Figure 1a. Five types of laminate specimens were prepared in this experiment, including two glass fiber-reinforced laminates made of glass fibers combined with VE-1 and VE-2, designated as GFRP-1 and GFRP-2, respectively, and three carbon/glass fiber hybrid-reinforced laminates, designated as HFRP-1, HFRP-2, and HFRP-3, respectively. The average thickness of all laminates was maintained at approximately 4.2 mm, and the relevant parameters are summarized in Table 4.

After completion of resin infusion, the laminates were cured at room temperature for 24 h, followed by post-curing in an oven at 100 °C for 4 h. The lay-up configurations of the three carbon/glass hybrid laminates are schematically shown in Figure 1b. According to the relevant mechanical testing standards, the laminates were machined into specimens with specified dimensions using a computer numerical control (CNC) engraving machine supplied by Kemei Numerical Control and Laser Development Co., Ltd., as illustrated in Figure 1c. The edges of all specimens were polished with fine-grit sandpaper and cleaned with anhydrous ethanol to remove machining residues and surface contaminants. Subsequently, the specimen edges were sealed to reduce moisture ingress through cut edges and exposed fiber ends, thereby minimizing edge effects [29]. Specifically, the specimen edges were sealed by applying a polytetrafluoroethylene (PTFE) emulsion. After the PTFE coating was fully cured at room temperature, the specimens were dried in an oven at 50 °C for 48 h to remove possible residual moisture.

### 2.3. Moisture Preconditioning

Considering that polar-going vessels may pass through mid-/low-latitude or humid marine regions before entering high-latitude cold waters, composite structures may have already absorbed a certain amount of moisture before low-temperature service. To simulate this service-relevant pre-absorbed state, all specimens except the room-temperature reference group were moisture preconditioned prior to environmental conditioning via full immersion in deionized water at 22 ± 1 °C for 150 d, following ASTM D5229 [30]. The 150-day immersion period was not a fixed duration prescribed by ASTM D5229, but was selected in this study to obtain a relatively stable long-term moisture-conditioned state. Although some FRP composites may approach moisture saturation within a shorter period, the time required to reach a stable moisture state depends on the resin system, laminate thickness, fiber architecture, edge sealing, and immersion temperature. Considering the approximately 4.2 mm thickness of the laminates, the sealed specimen edges, and the relatively low immersion temperature of 22 °C, a 150-day period was adopted to provide sufficient and consistent moisture exposure for all laminates before subsequent low-temperature exposure or FTC. Deionized water was used as a simplified conditioning medium to minimize additional variables associated with salt ions and salt crystallization. It should be noted that this medium cannot fully reproduce the chemical environment of seawater, where salinity and dissolved ions may further affect the resin–fiber interface and damage evolution. Therefore, the present protocol should be regarded as a moisture-assisted low-temperature/FTC conditioning procedure relevant to marine service, rather than a complete simulation of seawater exposure. During moisture preconditioning, the mass variation of the specimens was periodically monitored using a gravimetric method. Before each measurement, residual water on the specimen surface was gently removed, and the specimens were immediately weighed to minimize the influence of surface-adhered water on the measurement results. The moisture uptake was calculated using Equation (1).

(1) $$M_{t}=\frac{m_{t}-m_{0}}{m_{0}}\times 100\%$$

where $M_{t}$ is the moisture uptake after immersion time t, $m_{t}$ is the specimen mass after immersion time t, and $m_{0}$ is the initial specimen mass before moisture absorption.

In this study, moisture preconditioning was performed to provide a consistent moisture exposure history before subsequent environmental conditioning.

### 2.4. Environmental Conditioning Design

Previous studies have shown that FTC parameters for FRP composites are not standardized, with considerable variations in freezing temperature, thawing temperature, cycle duration, and number of cycles across different studies [18,31,32,33,34,35]. As shown in Table 5, FTC protocols reported for FRP composites vary greatly in temperature range, cycle number and cycle duration. Some studies used a large number of cycles, but each cycle generally lasted from several hours to 24 h. In the present study, the number of cycles was limited to three, while each cycle included a much longer freezing stage of 72 h at −50 °C followed by 24 h thawing in deionized water at 22 °C. Therefore, the three-cycle FTC protocol was designed to evaluate the early response and initial damage development of moisture-preconditioned laminates under prolonged low-temperature freezing and subsequent thawing, rather than to reproduce long-term Arctic service or predict service life.

On this basis, the low-temperature exposure in this study was set at −50 °C, and three environmental conditions were designed to compare the early residual responses of the laminates under different conditioning histories. Condition 1 was used as the room-temperature reference condition, in which the specimens were tested directly without moisture preconditioning. Condition 2 consisted of 150-day moisture preconditioning in deionized water at 22 °C followed by −50 °C exposure for 3 days. Condition 3 consisted of the same 150-day moisture preconditioning followed by three freeze–thaw cycles. In each cycle, the specimens were frozen at −50 °C for 3 days and then thawed/immersed in deionized water at 22 °C for 1 day.

The temperature of −50 °C was selected as a conservative low-temperature exposure level with reference to polar marine engineering practice. According to the IMO Polar Code, the Polar Service Temperature (PST) for ships operating in low air temperature should be at least 10 °C below the lowest Mean Daily Low Temperature (MDLT) for the intended area and season of operation [36]. In addition, the ABS Guide for Vessels Operating in Low Temperature Environments provides low-temperature material testing requirements, in which −50 °C is listed as an impact test temperature for certain steels used at a design service temperature of −30 °C [37]. Therefore, −50 °C was adopted in this study to evaluate the early response of moisture-preconditioned laminates under a severe low-temperature condition, rather than to represent all Arctic marine service temperatures.

**Figure 2:**
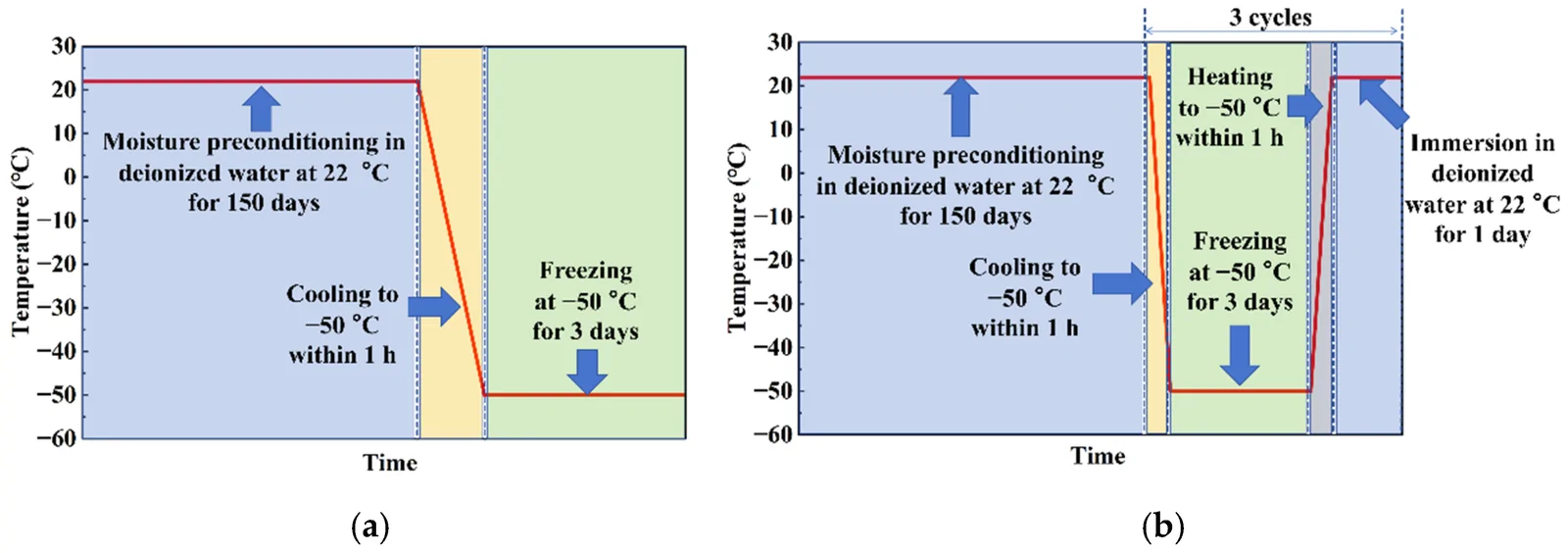
Schematic diagrams of the environmental conditioning procedures: (**a**) Condition 2, 150-day moisture preconditioning followed by −50 °C exposure; and (**b**) Condition 3, 150-day moisture preconditioning followed by FTC.

With this conditioning design, the differences between Condition 1 and Condition 2 reflect the combined influence of 150-day moisture preconditioning and subsequent −50 °C exposure. Similarly, the differences between Condition 1 and Condition 3 reflect the combined influence of 150-day moisture preconditioning and subsequent FTC. The present study was intended to compare the residual mechanical and damage responses of different laminate systems under these designed environmental histories. Since a separate moisture-only condition was not included, the individual effect of moisture absorption alone could not be quantitatively separated from the subsequent low-temperature exposure or FTC. This limitation was considered when interpreting the DMA, mechanical, and damage characterization results. The detailed conditioning procedure is shown in Figure 2.

### 2.5. Test Methods

#### 2.5.1. Dynamic Mechanical Analysis (DMA)

Dynamic mechanical analysis (DMA) was conducted using a DMA 8000 dynamic mechanical analyzer (PerkinElmer, Shelton, CT, USA) with reference to ASTM D7028 [38]. Laminate specimens with dimensions of 42 mm × 10 mm × 4 mm were tested in three-point bending mode over a temperature range of 25–200 °C at a heating rate of 5 °C/min. During the test, a sinusoidal load was applied at a frequency of 1 Hz with a strain amplitude of 0.05%, and the static force was set to 2 N. The frequency of 1 Hz and the heating rate of 5 °C/min were selected with reference to ASTM D7028 and commonly used DMA testing practice. These parameters were used to obtain comparable glass transition temperature and relaxation behavior among different laminates after environmental conditioning, rather than to reproduce the actual thermal or mechanical loading history during Arctic service. The glass transition temperature ($T_{g}$) was determined from the peak temperature of the loss factor (tanδ) curve, and each value was reported as the average of three replicate specimens.

#### 2.5.2. Mechanical Tests

Tensile, compressive, flexural, in-plane shear, and interlaminar shear tests were performed using an LE055 universal testing machine with a load capacity of 100 kN, manufactured by Shanghai Lishi Testing Instrument Co., Ltd. (Shanghai, China) During testing, the specimen temperature was maintained at approximately 25 °C. Therefore, the mechanical results reported in this study represent the residual properties of the laminates after environmental conditioning, rather than their in situ mechanical behavior at −50 °C. The loading rates used in the mechanical tests were selected according to the relevant ASTM test methods and commonly used quasi-static testing conditions. These rates were adopted to ensure repeatable residual-property evaluation and comparability among different materials and environmental conditions, rather than to simulate the actual loading rate of composite structures during Arctic service. For each laminate under each environmental condition, six replicate specimens were tested, and the average values of the valid results were reported as the final mechanical properties. When strain measurement was required, KYOWA strain gauges (model: KFGS-5-120-C1-11 Kyowa Electronic Instruments Co., Ltd., Chofu, Tokyo, Japan) bonded with CC-33A room-temperature quick-curing adhesive were used in this study.

The tensile properties of the laminates were determined according to GB/T 1447-2005 [39], the standard test method for tensile properties of fiber-reinforced polymer matrix composites. Tensile strain was measured using an extensometer, from which the tensile modulus was calculated. The crosshead speed was set to 2 mm/min.

The compressive properties of the laminates were determined in accordance with ASTM D6641 [40]. Before testing, end tabs were bonded to the compression specimens using AB epoxy adhesive and allowed to cure completely before being mounted in the fixture. The tabs had the same width as the compression specimens, namely 13 mm, and a length of 6.35 mm. Compressive strain was measured using strain gauges bonded at the central gauge region of the specimen, from which the compressive modulus was calculated. The crosshead speed was set to 2 mm/min.

The flexural properties of the laminates were tested in accordance with ASTM D7264 [41]. Flexural deflection was measured using a dial indicator, and the flexural modulus was calculated accordingly. The span-to-thickness ratio was set to 16:1, and the crosshead speed was set to 2 mm/min during flexural testing.

The in-plane shear properties of the laminates were determined in accordance with ASTM D7078 [42] using V-notched rail shear specimens. For this test, two strain gauges were bonded at the center of the test section between the two notch tips and oriented at +45° and −45° relative to the loading axis to measure the shear strain. The crosshead speed was set to 2 mm/min.

The interlaminar shear properties of the laminates were determined in accordance with ASTM D2344 [43] using short-beam specimens. The support span was set to 17 mm, corresponding to approximately four times the average specimen thickness. The specimen length of 30 mm provided sufficient overhang beyond the supports during testing. The crosshead speed was set to 1 mm/min.

For the compression-after-impact (CAI) tests, low-velocity impact damage was first introduced using a DIT752E impact testing machine manufactured by Shenzhen Wance Testing Machine Co., Ltd., Shenzhen, Guangdong, China. Low-velocity impact damage was introduced with reference to ASTM D7136/D7136M [44], and the subsequent CAI tests were conducted with reference to ASTM D7137/D7137M [45]. The impact energy was selected with reference to the thickness-normalized impact energy commonly used in ASTM D7136/D7136M. Since the average laminate thickness was approximately 4.2 mm, the impact energy estimated from 6.7 J/mm was approximately 28.1 J. Therefore, 30 J was adopted as a practical impact energy level for all laminate systems. The impactor mass and diameter were 9.5 kg and 16 mm, respectively. The same impact energy, impactor mass, and impactor diameter were used for all laminates to provide a common external impact condition for comparing their residual post-impact compressive performance. The selected energy level was intended to introduce a measurable low-velocity impact damage state suitable for subsequent CAI testing, rather than to produce identical damage areas or identical damage severity in all material systems. In the present work, the post-impact response was evaluated mainly through CAI strength and strength retention after the same impact condition, and the specimens were subjected to compression testing directly after impact. The impact height was determined using Equation (2).

(2) $$H=\frac{E}{m\cdot g}$$

where *H* represents the impact height (m), *E* is the impact energy (J), *m* is the impactor mass (kg), and *g* is the gravitational acceleration, taken as 9.81 m/s^2^.

After impact, the damaged specimens were subjected to compression testing at a crosshead speed of 1.25 mm/min. The test was continued until the maximum load was reached and terminated when the load decreased by approximately 30% from the maximum load.

#### 2.5.3. Scanning Electron Microscopy (SEM)

Scanning electron microscopy (SEM) observations were performed using a JSM-IT800 field-emission scanning electron microscope (JEOL Ltd., Akishima, Tokyo, Japan) to examine the microstructural damage features of the laminates. Two types of specimens were prepared for SEM analysis. Interlaminar shear specimens were used to observe the microstructure and interfacial damage in the interlaminar regions of the laminates, whereas fracture surfaces after tensile testing were examined to analyze the fracture morphology and failure features. Before observation, all specimens were sputter-coated with a thin platinum layer to improve surface conductivity.

#### 2.5.4. Fourier Transform Infrared Spectroscopy (FTIR)

Fourier transform infrared spectroscopy (FTIR) was used to examine possible FTIR-detectable changes in the main chemical functional groups of the resin matrix during environmental exposure. Prior to testing, approximately 15 mg of powder was gently scraped from the resin-rich regions of the laminate surface, avoiding fibers and sealed edges, thoroughly mixed with potassium bromide (KBr), and pressed into pellets. FTIR spectra were collected using an iN10-iS50 spectrometer (Thermo Scientific, Waltham, MA, USA) over the wavenumber range of 400–4000 cm^−1^. Each spectrum was obtained by averaging 60 scans at a spectral resolution of 4 cm^−1^.

## 3. Results and Discussion

It should also be noted that all mechanical tests were conducted at approximately 25 °C after environmental conditioning. Therefore, the following discussion focuses on residual mechanical performance and damage evolution after conditioning, rather than on the in situ mechanical response of the laminates at −50 °C. To eliminate the interference of resin-system variations and better isolate the effect of carbon/glass hybridization, the mechanical responses of the laminates were analyzed using a paired-comparison approach. Comparisons between HFRP-1 and GFRP-1 were used to evaluate the influence of CF-1/GF hybridization within the VE-1 resin system, whereas comparisons among HFRP-2, HFRP-3 and GFRP-2 were used to reveal the role of CF-2/GF hybridization in the mechanical behavior and environmental stability of the VE-2-based laminates. It should be noted that the Condition 1 datasets, including FTIR, DMA, and quasi-static mechanical properties of the five laminates, were previously reported by Zhou et al. [29] and are used here as the unconditioned reference baseline. The present study adds the Condition 2 and 3 datasets together with CAI results for all three conditions. The differences between Conditions 2 and 3 were therefore discussed as responses to two different environmental histories, rather than as the isolated effect of thermal cycling alone. Since marine composite structures are susceptible to low-velocity impact events during service, CAI testing was incorporated in addition to conventional tensile, compressive, flexural, in-plane shear and interlaminar shear tests. The CAI response provides an assessment of the residual compressive load-bearing capacity and damage tolerance of laminates after impact-induced damage.

### 3.1. FTIR Analysis

**Figure 3:**
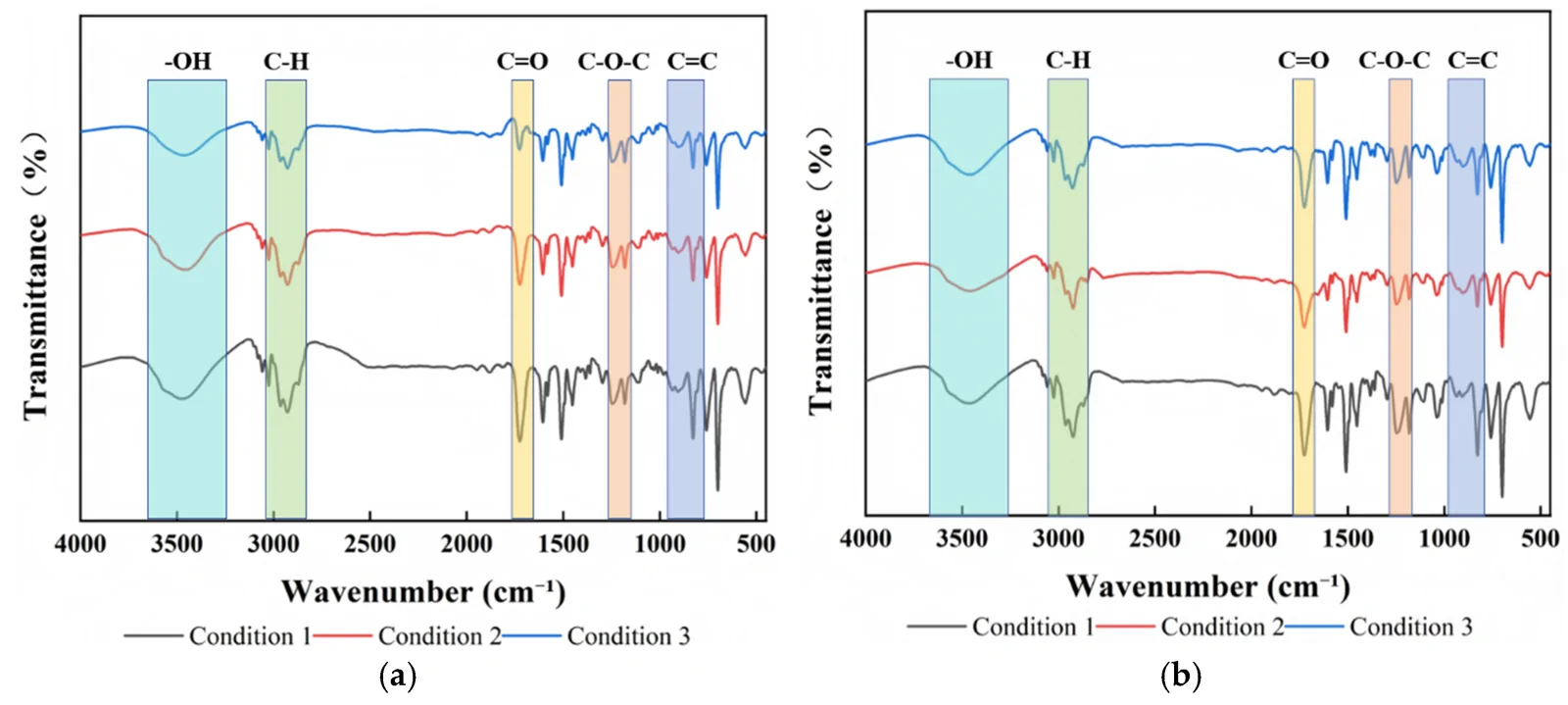
Fourier transform infrared (FTIR) spectra of (**a**) GFRP-1 and (**b**) GFRP-2 under Condition 1, Condition 2, and Condition 3.

Figure 3a,b present the FTIR spectra of GFRP-1 and GFRP-2 under different environmental conditions. Under Condition 1, the major characteristic absorption peaks of the two laminates appeared at similar positions with only minor differences in intensity, suggesting that the two resin systems had broadly similar chemical structures detectable by FTIR. Under all environmental conditions, the characteristic absorption peaks, including the O–H stretching vibration peak (~3400 cm^−1^), aromatic C-H stretching vibration peaks (3060 and 3026 cm^−1^), aliphatic C-H stretching vibration peaks (2964–2873 cm^−1^), ester carbonyl (C=O) stretching vibration peak (~1726 cm^−1^), aromatic ring skeletal vibration peaks (1606–1454 cm^−1^), and aromatic C-H out-of-plane bending vibration peaks (~758 and 700 cm^−1^), remained at nearly identical positions, while only minor variations in peak intensity were observed. Furthermore, no obvious new absorption bands or systematic peak shifts were observed after environmental conditioning. This indicates that no pronounced changes in the main characteristic absorption bands of the vinyl ester matrix were detected by FTIR under the investigated conditions. Similar observations were reported by Zhou et al. in studies on the environmental aging of FRP composites [29]. Since measurable changes in mechanical properties were still observed, the property variations were more likely associated with interfacial deterioration, microcrack development, and physical damage accumulation.

### 3.2. Dynamic Mechanical Analysis

**Figure 4:**
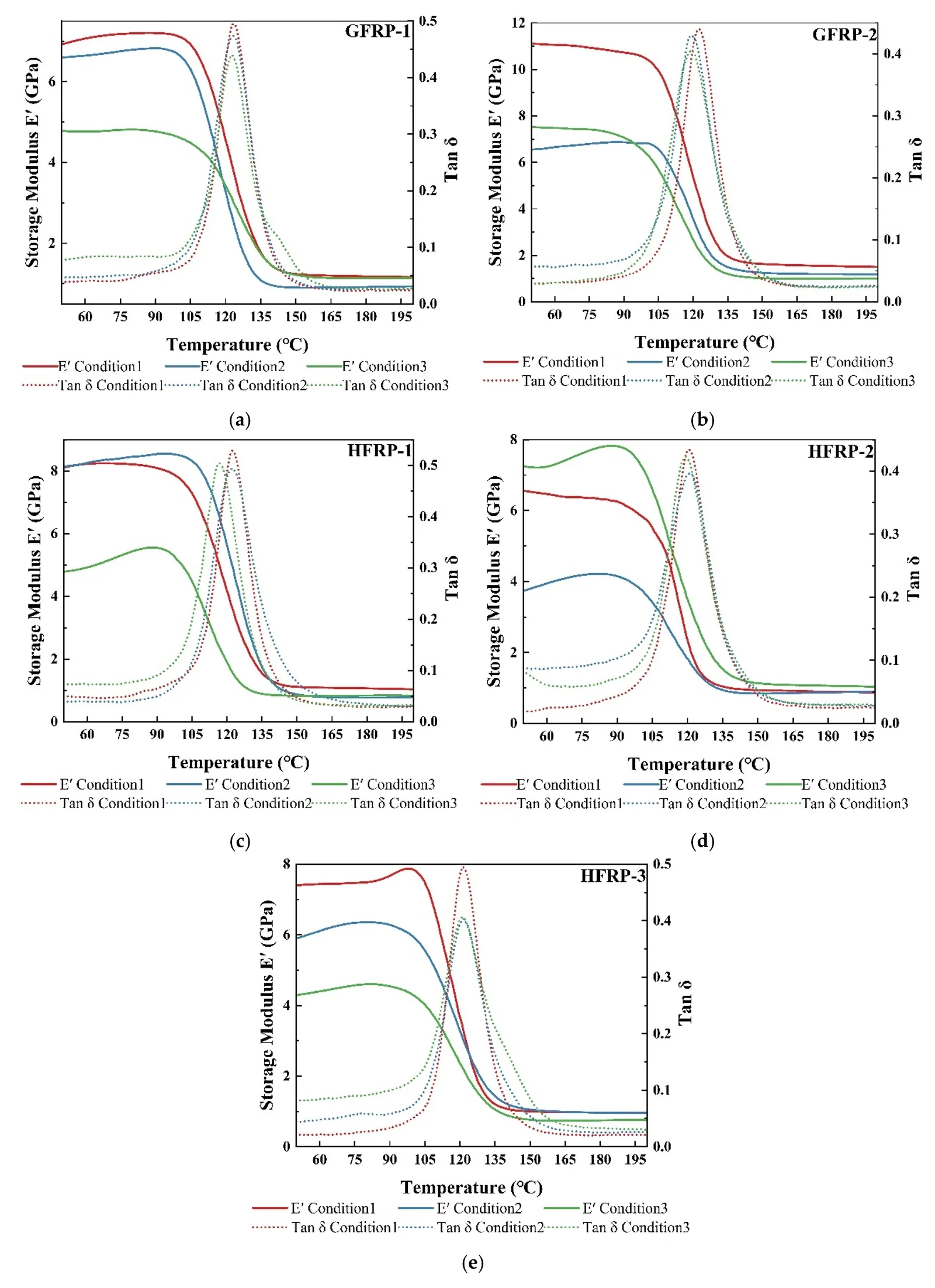
Dynamic mechanical analysis (DMA) results of (**a**) GFRP-1, (**b**) GFRP-2, (**c**) HFRP-1, (**d**) HFRP-2, and (**e**) HFRP-3 under three environmental conditions.

Figure 4a–e present the dynamic mechanical analysis (DMA) results of the five laminates under different environmental conditions, including the storage modulus ($E^{\prime }$) and damping factor (tanδ) curves. The glass transition temperature (${Τ}_{g}$) was identified from the peak temperature of the (tanδ) curve, and the corresponding ${Τ}_{g}$ and full width at half maximum (FWHM) values are summarized in Table 6. The FWHM of the tanδ peak is commonly used to describe the breadth of the relaxation time spectrum and the thermomechanical heterogeneity of the material. A larger FWHM generally indicates a broader distribution of relaxation behavior. All laminates exhibited typical DMA behavior of thermosetting polymer composites, with $E^{\prime }$ decreasing gradually with increasing temperature and dropping sharply near ${Τ}_{g}$. Because the overall $E^{\prime }$ and tanδ profiles were similar among the laminates, the following discussion focuses mainly on ${Τ}_{g}$ and FWHM, which are more sensitive to changes in thermomechanical relaxation behavior.

These results suggest that environmental exposure modified the local relaxation behavior and thermomechanical heterogeneity of the laminates, although the overall DMA response remained similar among the different conditions. Compared with the GFRP laminates, the ${Τ}_{g}$ response of the hybrid laminates was strongly dependent on the carbon/glass hybrid architecture. The ${Τ}_{g}$ variations between Conditions 2 and 3 were less than 1 °C for both GFRP-1 and GFRP-2, whereas more noticeable changes were observed in the hybrid laminates. HFRP-1 showed the largest reduction in ${Τ}_{g}$, decreasing by 0.54 °C after Condition 2 and by 5.54 °C after Condition 3 relative to Condition 1. Since only minor changes in ${Τ}_{g}$ were observed for GFRP-1 after both environmental treatments, the pronounced reduction in ${Τ}_{g}$ observed in HFRP-1 is unlikely to be attributed solely to changes in the resin matrix. Instead, it is more likely associated with the unique carbon/glass hybrid architecture. During FTC, the freezing of absorbed moisture and the thermal mismatch among carbon fibers, glass fibers, and the resin matrix may generate localized stresses near the fiber/matrix interfaces and carbon/glass transition regions, contributing to possible interfacial and interlaminar damage. These effects may change the local thermomechanical relaxation response of the laminate, which is consistent with the observed decrease in ${Τ}_{g}$. This result indicates a higher thermomechanical sensitivity of HFRP-1 to the designed FTC history. The interfacial debonding and interlaminar damage observed in the subsequent SEM analysis are also consistent with this interpretation. In contrast, the variations in ${Τ}_{g}$ HFRP-2 and HFRP-3 were relatively small. Under Condition 2, the changes in ${Τ}_{g}$ were negligible compared with the Condition 1 and were substantially smaller than the approximately 3 °C reduction observed for GFRP-2. This result suggests that the CF-2/GF hybrid architecture showed a relatively stable thermomechanical relaxation response during low-temperature exposure, which may explain the limited change in ${Τ}_{g}$. Under Condition 3, the ${Τ}_{g}$ values of HFRP-2 and HFRP-3 decreased only slightly by approximately 0–2 °C, whereas no further reduction was observed for GFRP-2. This suggests that FTC still affected the local thermomechanical response of the CF-2/GF hybrid laminates, leading to a slight decrease in ${Τ}_{g}$, although the effect was considerably less pronounced than that observed for HFRP-1. This observation is consistent with the subsequent mechanical test results, further indicating that HFRP-1 exhibited a higher sensitivity to FTC, whereas HFRP-2 and HFRP-3 maintained relatively stable performance.

Examination of the FWHM results shows that both GFRP laminates exhibited an increase in peak width after environmental conditioning, indicating an increase in structural heterogeneity. For GFRP-1, the FWHM increased from 16.79 °C under Condition 1 to 19.27 °C after Condition 2 and then decreased slightly to 17.97 °C after Condition 3. Although the value after Condition 3 remained higher than that under Condition 1, it was lower than that after Condition 2. This result suggests that the relaxation spectrum of GFRP-1 was broadened after the designed environmental histories, but Condition 3 did not further increase the dispersion of relaxation behavior compared with Condition 2. In contrast, GFRP-2 exhibited a different response, with the FWHM increasing by approximately 3 °C and 6 °C under Conditions 2 and 3, respectively, relative to Condition 1. This finding suggests that the Condition 3 environmental history further broadened the relaxation time distribution of the VE-2/GF system, resulting in increased structural heterogeneity and a wider distribution of local molecular environments. Compared with Condition 1, the FWHM of HFRP-1 increased markedly under both Conditions 2 and 3, with a greater increase than that observed for GFRP-1 fabricated with the same VE-1 resin system. This result indicates a higher sensitivity of the CF-1/GF hybrid laminate to environmental exposure, leading to a broader distribution of relaxation behavior. Although the FWHM of HFRP-1 under Condition 3 was slightly lower than that under Condition 2, it remained higher than that of the Condition 1. This suggests that FTC did not further broaden the relaxation time distribution, while the increase in structural heterogeneity induced by environmental conditioning was still retained. For the VE-2 system, the FWHM of HFRP-2 increased from 18.96 °C under Condition 1 to 24.48 °C under Condition 2 and then decreased to 21.28 °C under Condition 3. This result indicates that the Condition 2 environmental history noticeably broadened the relaxation time distribution, whereas the dispersion of relaxation behavior was partially reduced after FTC, although it remained higher than that of the Condition 1. In contrast, the FWHM of HFRP-3 further increased to 25.96 °C under Condition 3, exceeding those of both GFRP-2 and HFRP-2, suggesting a more pronounced broadening of the relaxation spectrum under FTC. This observation indicates that, in addition to the resin system, the stacking configuration also plays an important role in determining the thermomechanical response of the laminate. Owing to the different distribution of carbon/glass interfaces in HFRP-3, the moisture-preconditioned FTC history in Condition 3 may produce a broader distribution of local relaxation environments, resulting in a wider relaxation time spectrum and a higher degree of thermomechanical heterogeneity.

### 3.3. Mechanical Properties

The residual mechanical response of the laminates depends on the dominant load-bearing and failure mechanisms of each test. Tensile properties are mainly governed by the reinforcing fibers, whereas compressive, flexural, interlaminar shear, and CAI properties are more sensitive to matrix damage, fiber–matrix interfacial integrity, interlaminar defects, and local instability. For the HFRP laminates, the different thermal expansion behavior of carbon fibers, glass fibers, and the resin matrix, together with the freezing/thawing of absorbed moisture, may promote local matrix/interface-related damage during the designed FTC history. The overall residual mechanical response is summarized in Table 7.

#### 3.3.1. Tensile Properties

**Figure 5:**
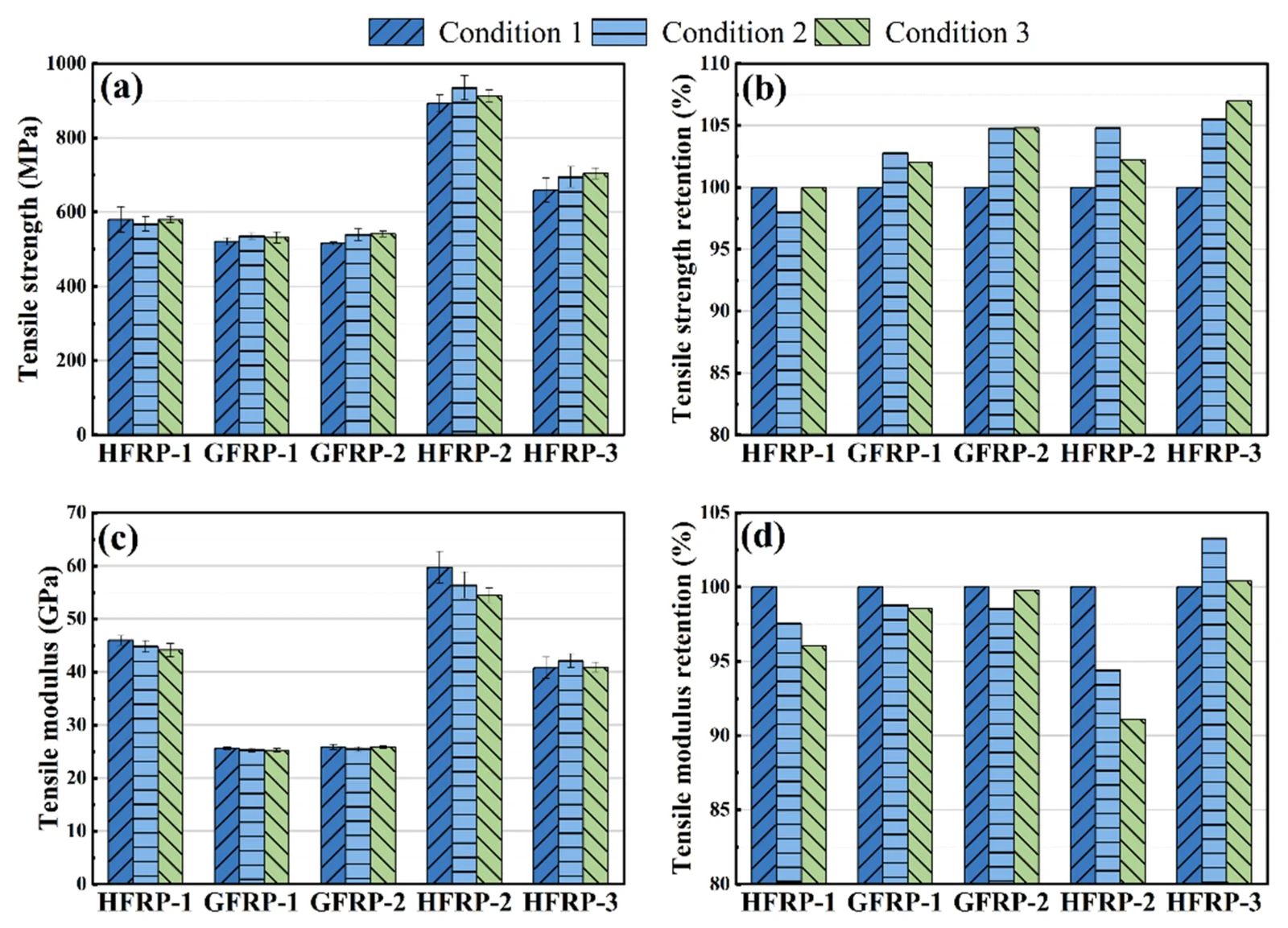
Tensile properties of GFRP and HFRP laminates under different conditions: (**a**) tensile strength, (**b**) tensile strength retention, (**c**) tensile modulus, (**d**) tensile modulus retention.

The tensile strength and strength retention of the GFRP and HFRP laminates are presented in Figure 5a,b. Under Condition 1, GFRP-1 and GFRP-2 exhibited comparable tensile strengths, indicating that the difference between the two vinyl ester resin systems had a limited influence on the tensile strength of the pure GFRP laminates. Compared with GFRP-1, the tensile strength of HFRP-1 increased by 11.2%, whereas HFRP-2 and HFRP-3 exhibited increases of 73.1% and 27.8%, respectively, relative to GFRP-2. These results demonstrate that the incorporation of carbon fibers can significantly enhance the tensile load-bearing capacity of the laminates. The extent of improvement depends not only on the carbon fiber volume fraction but also on the fiber type and the efficiency of load transfer at the fiber–matrix interface. After Condition 2, no obvious reduction in tensile strength was observed for any of the laminates, indicating that the combined history of moisture preconditioning followed by −50 °C exposure did not markedly impair the fiber-dominated load-bearing capability of the composites. Combined with the FTIR results, which showed no pronounced changes in the main characteristic absorption bands, the stable tensile strength suggests that the fiber-dominated load-bearing capability was largely preserved after Condition 2. Under Condition 3, the tensile strength variations of GFRP-1 and GFRP-2 remained within 5% of Condition 1, suggesting that the fiber-dominated tensile performance of the pure GFRP laminates was largely retained after moisture preconditioning followed by FTC. This observation is consistent with the findings reported by Hasan et al. [18]. Compared with the GFRP laminates, the HFRP laminates showed relatively larger fluctuations in tensile strength under Condition 3. Because carbon fibers and glass fibers have different thermal expansion behavior, repeated temperature changes during FTC may lead to local thermal mismatch near the carbon/glass and fiber/matrix interfaces [46,47]. This effect may partly explain the relatively larger tensile strength fluctuations observed in the HFRP laminates after Condition 3. For HFRP-1, although DMA showed a ${Τ}_{g}$ decrease of approximately 5.5 °C after Condition 3, its residual tensile strength remained nearly unchanged. This difference suggests that the DMA response was more sensitive to thermomechanical relaxation and possible matrix/interface-related changes, whereas the tensile strength measured at approximately 25 °C was still mainly governed by the continuous reinforcing fibers. This interpretation is consistent with the tensile fracture morphologies in, where fiber fracture remained the dominant failure feature, and the observed interfacial debonding was mainly localized.

Figure 5c,d present the tensile modulus and modulus retention of the GFRP and HFRP laminates. Under Condition 1, the tensile moduli of GFRP-1 and GFRP-2 were 25.59 GPa and 25.86 GPa, respectively, indicating only a marginal difference between the two laminates. Since both laminates were reinforced with glass fibers and the stiffness difference between the VE-1 and VE-2 resin systems was relatively small, their overall tensile moduli remained at comparable levels. The incorporation of carbon fibers led to a substantial increase in the tensile modulus of the laminates. Compared with GFRP-1, the tensile modulus of HFRP-1 increased by 79.3%, while HFRP-2 and HFRP-3 exhibited increases of 130.4% and 57.7%, respectively, relative to GFRP-2. These results demonstrate that the introduction of high-modulus carbon fibers can markedly increase the tensile stiffness of FRP laminates. Furthermore, the differences in modulus enhancement indicate that the tensile stiffness of hybrid laminates is influenced not only by the carbon fiber volume fraction but also by the stacking configuration. After exposure to Conditions 2 and 3, only minor variations in tensile modulus were observed for GFRP, HFRP-1, and HFRP-3, indicating a relatively stable tensile stiffness under environmental exposure. In contrast, the tensile modulus of HFRP-2 decreased by approximately 5.6% under Condition 2 and further declined to 8.8% below the Condition 1 value under Condition 3. The decrease in HFRP-2 tensile modulus may be related to its relatively high carbon fiber content and stacking configuration, which made the tensile stiffness more sensitive to interface-related load transfer after environmental conditioning.

#### 3.3.2. Compressive Properties

**Figure 6:**
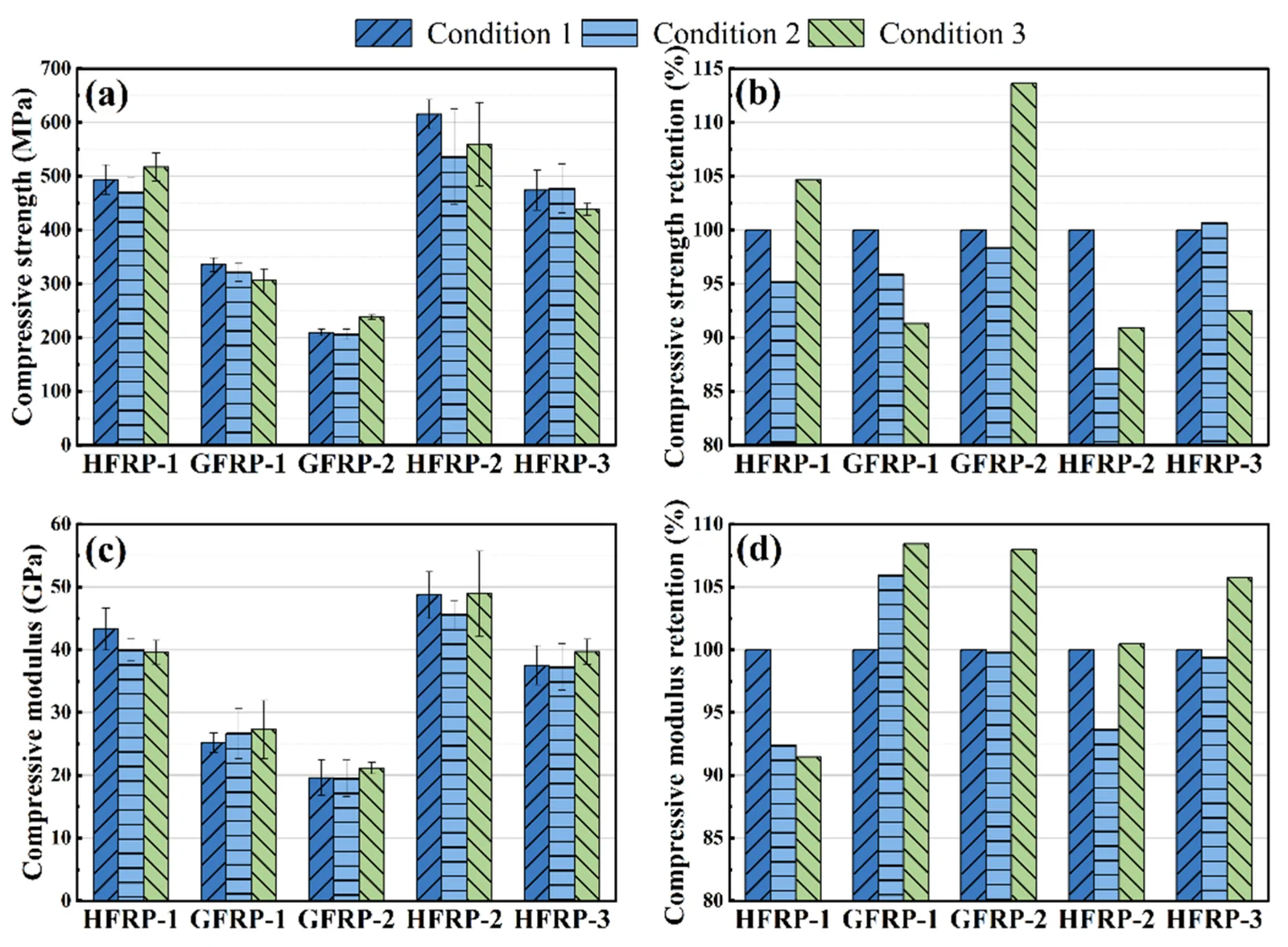
Compressive properties of GFRP and HFRP laminates under different environmental conditions: (**a**) compressive strength, (**b**) compressive strength retention, (**c**) compressive modulus, (**d**) compressive modulus retention.

Figure 6a,b present the compressive strength and strength retention of the GFRP and HFRP laminates. Under Condition 1, the compressive strength of GFRP-1 was 60.2% higher than that of GFRP-2. Since compressive behavior is generally more sensitive to matrix characteristics and fiber–matrix interfacial integrity than tensile behavior, the superior compressive performance of GFRP-1 is likely associated with the more effective load-transfer capability and stronger interfacial constraint of the VE-1/GF system. This interpretation is further supported by the higher ILSS of GFRP-1 and the relatively stable thermomechanical behavior revealed by DMA. Compared with GFRP-1, the compressive strength of HFRP-1 increased by 47.25%, whereas HFRP-2 and HFRP-3 exhibited improvements of 193.81% and 126.43%, respectively, relative to GFRP-2. The incorporation of carbon fibers significantly enhanced the compressive load-bearing capacity of the laminates. Owing to their higher stiffness and superior resistance to compressive instability, carbon fibers can carry a larger proportion of the applied load and effectively suppress fiber microbuckling and local instability, thereby resulting in a substantial improvement in compressive strength [48]. Furthermore, the higher compressive strength of HFRP-2 compared with HFRP-3 indicates that carbon fiber content and stacking configuration are also important factors governing the compressive performance of hybrid laminates. After Condition 2, most laminates retained their compressive strength, whereas HFRP-2 showed a moderate decrease of 12.8% relative to Condition 1. This suggests that the compressive response of HFRP-2 was more sensitive to the designed moisture-assisted low-temperature history than that of the other laminates. The stronger sensitivity of compressive strength can be attributed to the instability-related nature of compressive failure in fiber-reinforced laminates. Unlike tensile failure, compressive failure requires effective lateral support from the matrix and efficient shear transfer at the fiber–matrix interface to suppress local fiber microbuckling, kink-band formation, and premature splitting. Therefore, local matrix plasticization, interfacial weakening, or thermally induced microdefects after the designed environmental histories may weaken the constraint on fibers under compression, even when the same changes are not sufficient to cause an obvious decrease in tensile strength.

After Condition 3, the compressive strength of GFRP-1 decreased by 8.7%, whereas that of GFRP-2 showed an apparent increase of 13.6%, indicating distinct responses of the two VE/GF systems. The apparent increase in GFRP-2 may be associated with the residual post-curing of the VE-2 resin during long-term moisture preconditioning and subsequent environmental exposure. Zhou et al. [29] reported a slight increase in the compressive strength of similar vinyl ester resins after FTC and attributed this behavior to resin post-curing. In the present study, this effect may have helped maintain matrix constraint and local load transfer in GFRP-2, although the result should still be interpreted as a property-dependent response rather than direct environmental strengthening. Considering the FTIR results and the SEM evidence of localized interfacial and interlaminar damage, the observed difference may be associated with variations in interfacial integrity, microstructural defect evolution, and local structural stability during compression. For the hybrid laminates, HFRP-1 remained essentially stable, while HFRP-2 and HFRP-3 decreased by approximately 9.1% and 7.5%, respectively. These results suggest that compressive strength was more sensitive than compressive modulus to matrix/interface condition and local instability, as summarized in Table 7.

Figure 6c,d present the compressive modulus and modulus retention of the laminates. Consistent with the compressive strength results, GFRP-1 exhibited a higher compressive modulus than GFRP-2. The incorporation of carbon fibers significantly increased the compressive stiffness of the hybrid laminates, with HFRP-2 exhibiting the highest modulus among all specimens. This behavior can be primarily attributed to the substantially higher modulus of carbon fibers compared with glass fibers, together with the relatively high carbon fiber volume fraction in HFRP-2, which enabled a greater proportion of the compressive load to be carried by the carbon fiber plies. Compared with compressive strength, the compressive modulus was less affected by environmental exposure, and most laminates retained relatively high stiffness after Conditions 2 and 3. Previous studies have shown that environmental degradation preferentially affects fiber–matrix interfacial integrity, microcrack propagation, and local instability mechanisms, while exerting a comparatively limited influence on the overall stiffness of composite laminates [49]. Therefore, the changes in compressive strength observed in this study were generally more pronounced than those in compressive modulus, indicating that environmental exposure primarily altered the compressive failure process and local structural stability rather than significantly reducing the overall compressive stiffness of the laminates.

#### 3.3.3. Flexural Properties

**Figure 7:**
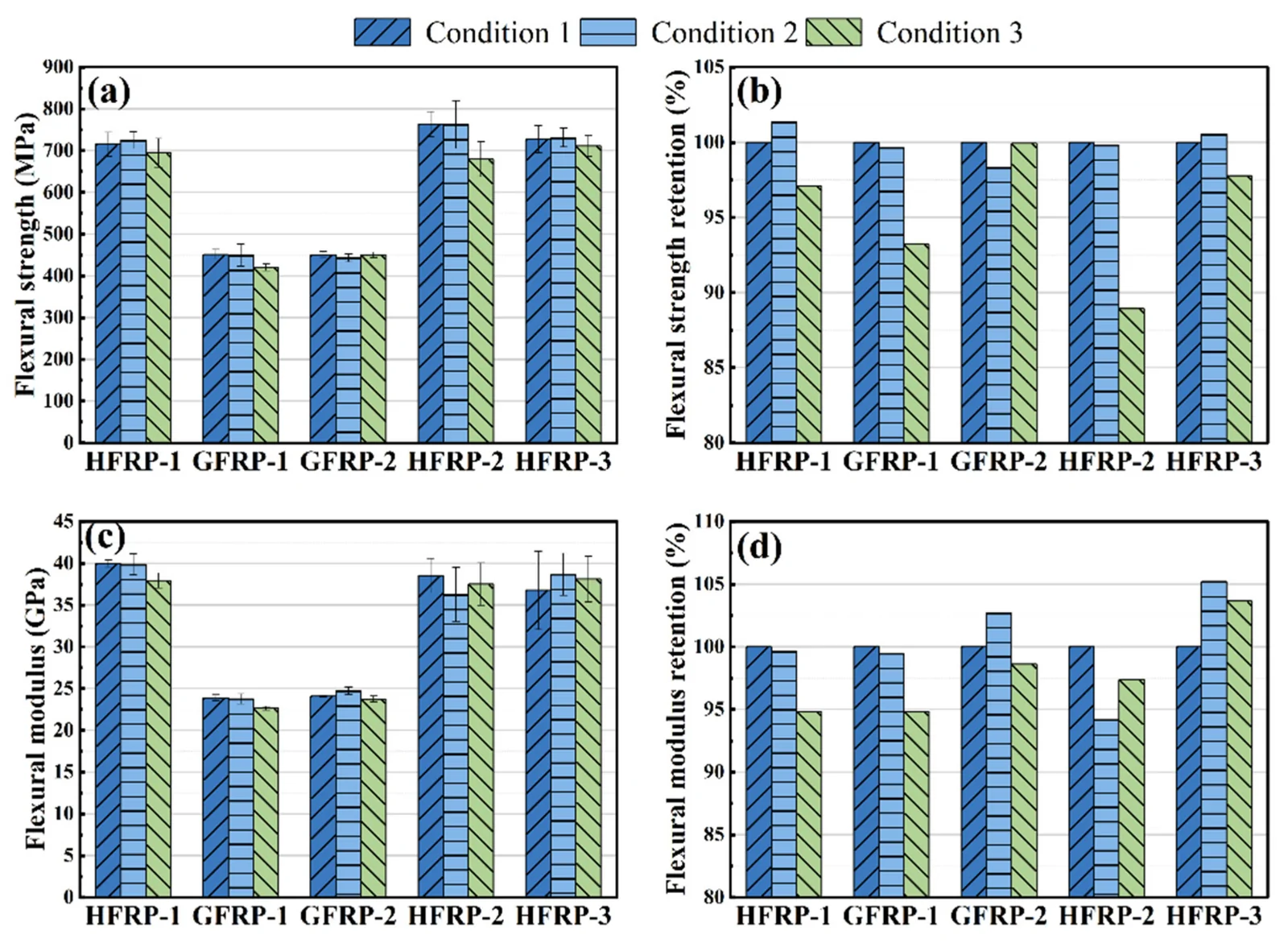
Flexural properties of GFRP and HFRP laminates under different environmental conditions: (**a**) flexural strength, (**b**) flexural strength retention, (**c**) flexural modulus, (**d**) flexural modulus retention.

Under flexural loading, the outer plies of the laminate are subjected to the highest tensile and compressive stresses, while interlaminar stresses through the thickness can markedly influence damage initiation and propagation. Consequently, flexural properties are particularly sensitive to laminate architecture, interfacial integrity, and environmentally induced damage [50]. Figure 7a,b present the flexural strength and strength retention of the laminates. Under Condition 1, GFRP-1 and GFRP-2 exhibited nearly identical flexural strengths, indicating that the difference between the two vinyl ester resin systems had a limited influence on the initial flexural performance of the pure GFRP laminates. Compared with GFRP-1, the flexural strength of HFRP-1 increased by 58.62%. Relative to GFRP-2, the flexural strengths of HFRP-2 and HFRP-3 increased by 69.56% and 61.46%, respectively. These results demonstrate that the incorporation of carbon fibers markedly improved the flexural load-bearing capacity of the laminates. Similar to the compressive strength results, after Condition 2, only minor changes in flexural strength were observed for all laminates, indicating that the flexural load-bearing capacity was largely retained after moisture preconditioning followed by −50 °C exposure. Overall, the hybrid structures maintained relatively stable flexural performance under the investigated low-temperature condition. After exposure to Condition 3, the flexural strength of GFRP-1 decreased by approximately 6.8% relative to Condition 1, whereas no obvious change was observed for GFRP-2. This trend is consistent with the compressive strength results, indicating distinct responses of the two VE/GF systems to FTC. Considering that no obvious chemical degradation was detected by FTIR, the reduction in flexural performance of GFRP-1 is more likely associated with changes in interfacial integrity and the accumulation of local damage rather than degradation of the resin matrix itself. These effects may reduce both compressive stability and flexural load-bearing capability. Among the hybrid laminates, HFRP-2 showed the largest reduction in flexural strength after Condition 3, with an approximately 11.1% decrease relative to Condition 1. This result indicates a higher sensitivity of this stacking configuration to flexural damage after Condition 3, as summarized in Table 7. For HFRP-2, the relatively high carbon fiber content and the clustered carbon/glass hybrid configuration may have promoted local stress redistribution around carbon/glass transition regions during bending, making flexural strength more sensitive to matrix/interface damage than the initial flexural modulus.

Figure 7c,d present the flexural modulus and modulus retention of the GFRP and HFRP laminates under different environmental conditions. Similar to the tensile modulus results, only a small difference in flexural modulus was observed between GFRP-1 and GFRP-2 under Condition 1, suggesting that the influence of the resin system on the overall flexural stiffness of the pure GFRP laminates was limited. Compared with the GFRP laminates, the incorporation of carbon fibers substantially increased the flexural modulus. The flexural modulus of HFRP-1 was approximately 67% higher than that of GFRP-1, while those of HFRP-2 and HFRP-3 were increased by about 60% and 52%, respectively, relative to GFRP-2. Notably, although HFRP-1 contained a lower carbon fiber volume fraction than HFRP-2, it exhibited a slightly higher flexural modulus. This observation indicates that flexural stiffness is governed not only by the carbon fiber content but also by the laminate stacking configuration. According to classical laminate theory, plies located farther from the neutral axis contribute more significantly to bending stiffness. Therefore, the through-thickness distribution of carbon fiber plies plays a critical role in determining the flexural modulus of hybrid laminates, and an optimized stacking sequence can more effectively exploit the high stiffness of carbon fibers. After Condition 2, the flexural modulus of most laminates changed within approximately ±6%, while HFRP-2 showed a moderate decrease of about 6%. Following Condition 3, the flexural modulus of HFRP-2 partially recovered relative to Condition 2 and remained only about 2.6% lower than the Condition 1 value, whereas the other laminates showed only minor changes. Overall, the flexural modulus was less sensitive than flexural strength, suggesting that the designed environmental histories had a greater influence on failure-related damage evolution than on the overall bending stiffness. This difference is reasonable because the initial flexural modulus mainly reflects the elastic bending stiffness before severe damage develops, whereas flexural strength is determined near final failure and is therefore more affected by local crack initiation, interface degradation, and damage propagation. As a result, localized damage may reduce flexural strength without causing a comparable decrease in the initial bending stiffness.

#### 3.3.4. In-Plane Shear Properties

**Figure 8:**
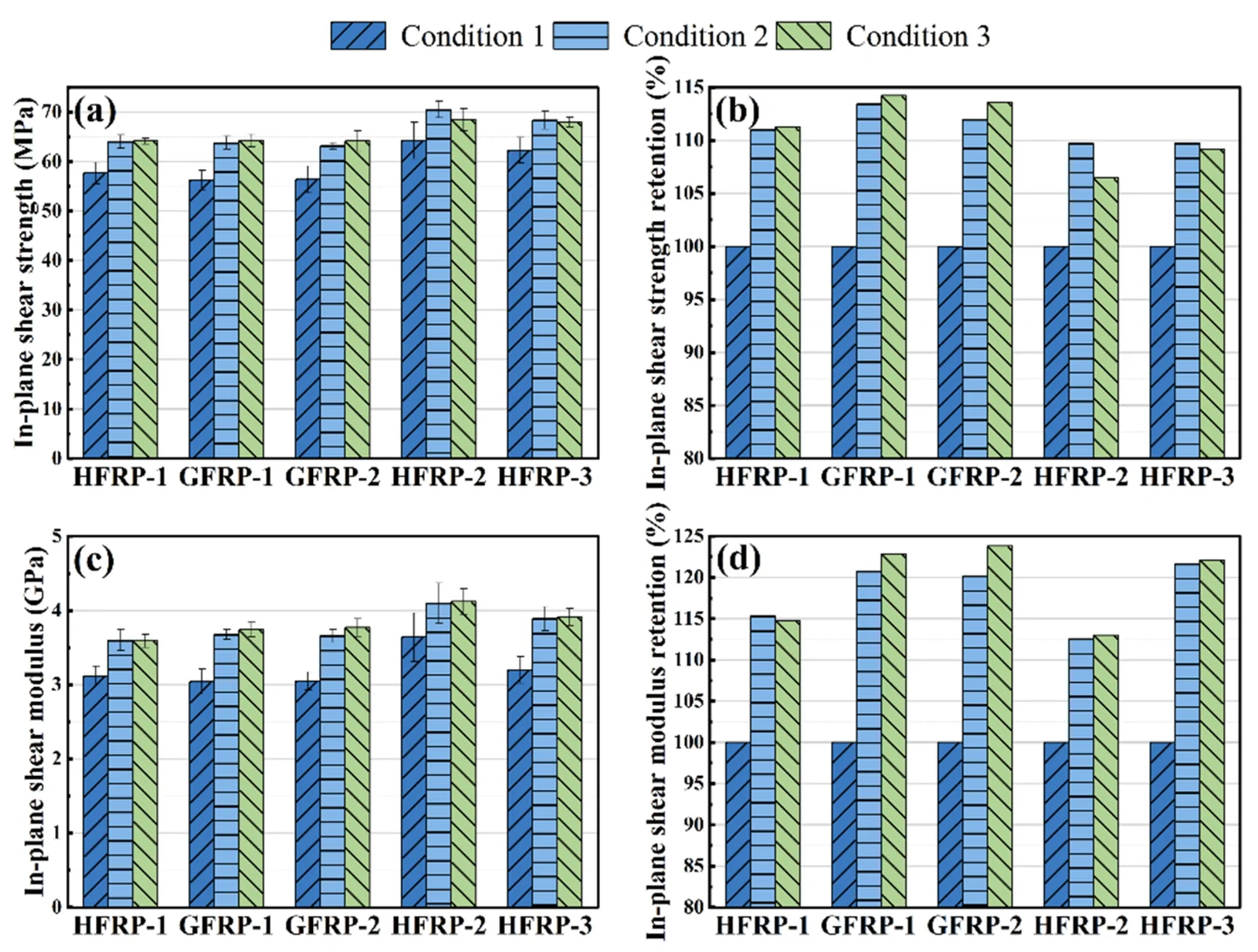
In-plane shear properties of GFRP and HFRP laminates under different environmental conditions: (**a**) in-plane shear strength, (**b**) in-plane shear strength retention, (**c**) in-plane shear modulus, (**d**) in-plane shear modulus retention.

The in-plane shear strength and strength retention of the GFRP and HFRP laminates are presented in Figure 8a,b. For laminates without ±45° plies, the in-plane shear response is primarily governed by matrix shear deformation and fiber–matrix interfacial load transfer. Consequently, the in-plane shear properties are more sensitive to the condition of the matrix and interface, whereas the contribution of the fiber axial properties is relatively limited [51]. Under Condition 1, GFRP-1 and GFRP-2 exhibited nearly identical in-plane shear strengths, with a difference of less than 1%, owing to the comparable mechanical properties of the VE-1 and VE-2 resin systems. In contrast, the in-plane shear strength of HFRP-1 was only 2.47% higher than that of GFRP-1, whereas HFRP-2 and HFRP-3 exhibited increases of 14.04% and 10.48%, respectively, relative to GFRP-2. These results suggest that the CF-2/GF hybrid architecture provided a more pronounced enhancement in in-plane shear performance, whereas the improvement achieved by the CF-1 system was relatively limited. Such differences may be associated not only with the carbon fiber content but also with variations in interfacial load-transfer efficiency among the different fiber–resin systems. After Condition 2, the in-plane shear strengths of both GFRP and HFRP laminates increased by approximately 9–14%, suggesting that no deterioration in residual in-plane shear strength was observed after Condition 2. Since in-plane shear is sensitive to matrix deformation and fiber–matrix load transfer, this increase may be partly related to residual post-curing of the vinyl ester resin, which could improve matrix constraint after long-term conditioning. Under Condition 3, the in-plane shear strengths remained close to those under Condition 2, with variations generally within 5%, indicating that the residual in-plane shear response was largely retained under the designed moisture-preconditioned FTC history.

Figure 8c,d present the in-plane shear modulus and modulus retention of the GFRP and HFRP laminates. A similar trend was observed between the in-plane shear modulus and in-plane shear strength under all environmental conditions. The similar evolution of in-plane shear strength and modulus suggests that the environmental conditions employed in this study did not substantially alter the deformation and load-transfer mechanisms governing the in-plane shear response. After Condition 2, the in-plane shear modulus increased for all laminates, with retention ratios ranging from approximately 112.6% to 121.7%. Only minor differences were observed between Conditions 2 and 3, indicating that the residual in-plane shear stiffness remained relatively stable under the designed environmental histories. Compared with tensile, compressive, and flexural properties, the in-plane shear response exhibited lower sensitivity to the designed environmental histories, indicating that the residual matrix-dominated shear deformation behavior was largely retained.

#### 3.3.5. Interlaminar Shear Properties

**Figure 9:**
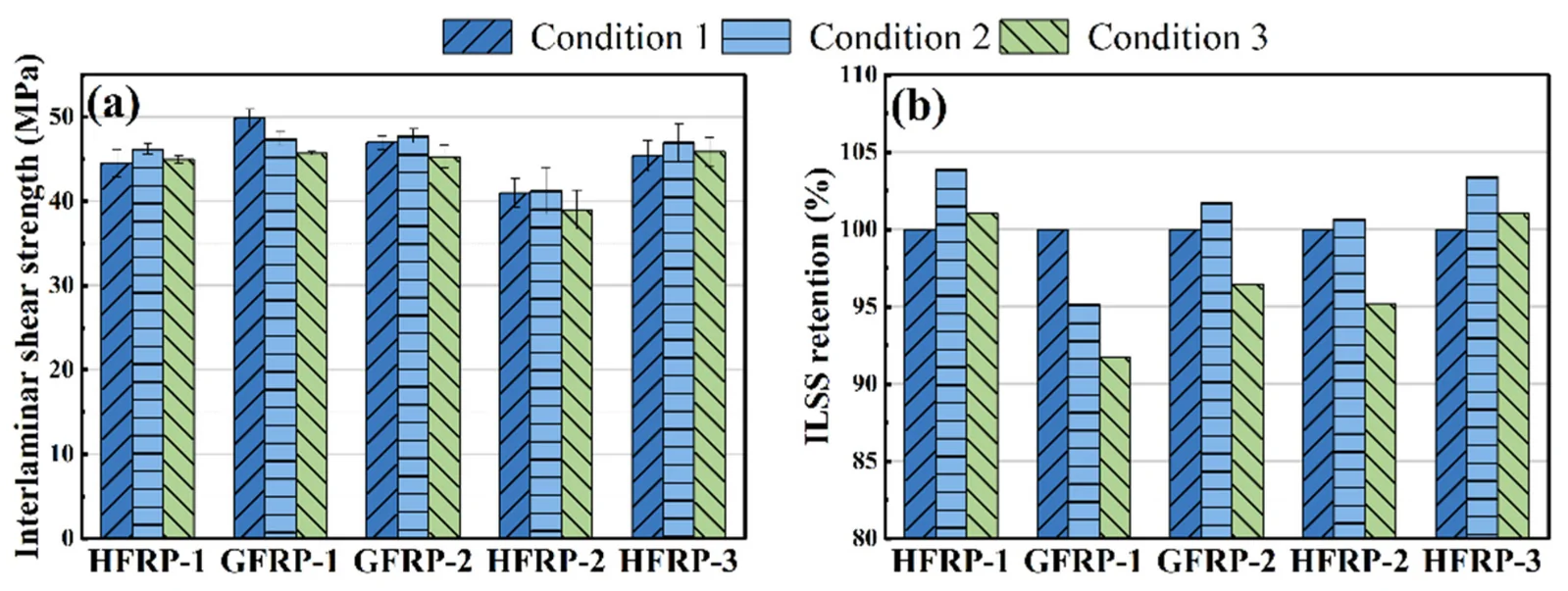
Interlaminar shear properties of GFRP and HFRP laminates under different environmental conditions: (**a**) interlaminar shear strength, (**b**) interlaminar shear strength retention.

The interlaminar shear strength (ILSS) and strength retention of the GFRP and HFRP laminates are presented in Figure 9a,b. ILSS is widely regarded as a key indicator of interfacial integrity and interlaminar bonding quality in composite laminates. Under Condition 1, the ILSS of GFRP-1 was approximately 6% higher than that of GFRP-2, suggesting that the VE-1/GF system possessed more effective interfacial load-transfer capability and stronger interlaminar bonding. This observation is consistent with the previously observed trends in compressive strength and compressive modulus, further indicating that the VE-1 system provided more effective interfacial constraint within the laminate. Under Condition 1, the ILSS of HFRP-1 was approximately 10.7% lower than that of GFRP-1, while HFRP-2 and HFRP-3 exhibited reductions of about 12.7% and 3.3%, respectively, relative to GFRP-2. Unlike the tensile, compressive, and flexural properties, the ILSS did not increase monotonically with increasing carbon fiber content and even decreased in some hybrid laminates. Notably, HFRP-3 exhibited a higher ILSS than HFRP-2. Because ILSS is highly sensitive to interfacial regions, the higher ILSS of HFRP-3 than HFRP-2 suggests that the distribution and bonding quality of carbon/glass interfaces are more important than carbon fiber content alone. Therefore, carbon/glass hybridization does not necessarily improve interlaminar shear performance, and its effect depends strongly on stacking configuration and interface quality. This interpretation is consistent with Monjon et al. [26], who reported that heterogeneous carbon/glass interfaces can adversely affect interlaminar shear performance.

Under Condition 2, only minor variations in ILSS were observed for all laminates, indicating that the interlaminar load-transfer capability was largely retained. More apparent changes were observed after Condition 3, with ILSS reductions of approximately 2.3–5.4% relative to Condition 2. This result is consistent with the interface-sensitive nature of ILSS and the interfacial damage features discussed in Section 3.4. The ILSS results can be understood from the localized and interface-sensitive stress state of the short-beam shear test. Although ILSS is reported as a strength value, failure in the short-beam test is strongly influenced by interlaminar stress transfer, fiber–matrix bonding, matrix cracking, and local damage initiation. Moisture absorbed in the matrix or along fiber–matrix interfaces can reduce interfacial resistance and promote local debonding. Subsequent freezing/thawing may further contribute to local stress concentration through thermal mismatch and moisture-related expansion.

#### 3.3.6. Compression After Impact (CAI) Properties

Compression after impact (CAI) is an important indicator for evaluating the damage tolerance and residual load-bearing capacity of composite laminates. Unlike conventional mechanical properties such as tensile, compressive, and flexural performance, CAI is highly sensitive to impact-induced delamination, interfacial damage, and subsequent damage propagation. For marine composite structures, where low-velocity impacts, environmental aging, and service loads may coexist, CAI provides a useful assessment of residual structural integrity and damage tolerance after environmental conditioning [52,53]. Recent studies on aged fiber-reinforced polymer composite pipes have also shown that environmental aging can affect low-velocity impact response and damage behavior, suggesting that impact-related performance should be evaluated after environmental conditioning [54]. In this study, the same impact energy, impactor mass, and impactor diameter were used for all laminates, and CAI strength and strength retention were used to compare their residual post-impact compressive performance after environmental conditioning. CAI strength provides a direct measure of the residual compressive load-bearing capacity and damage stability of impacted specimens, and can therefore reflect the environmental sensitivity of different laminate systems.

**Figure 10:**
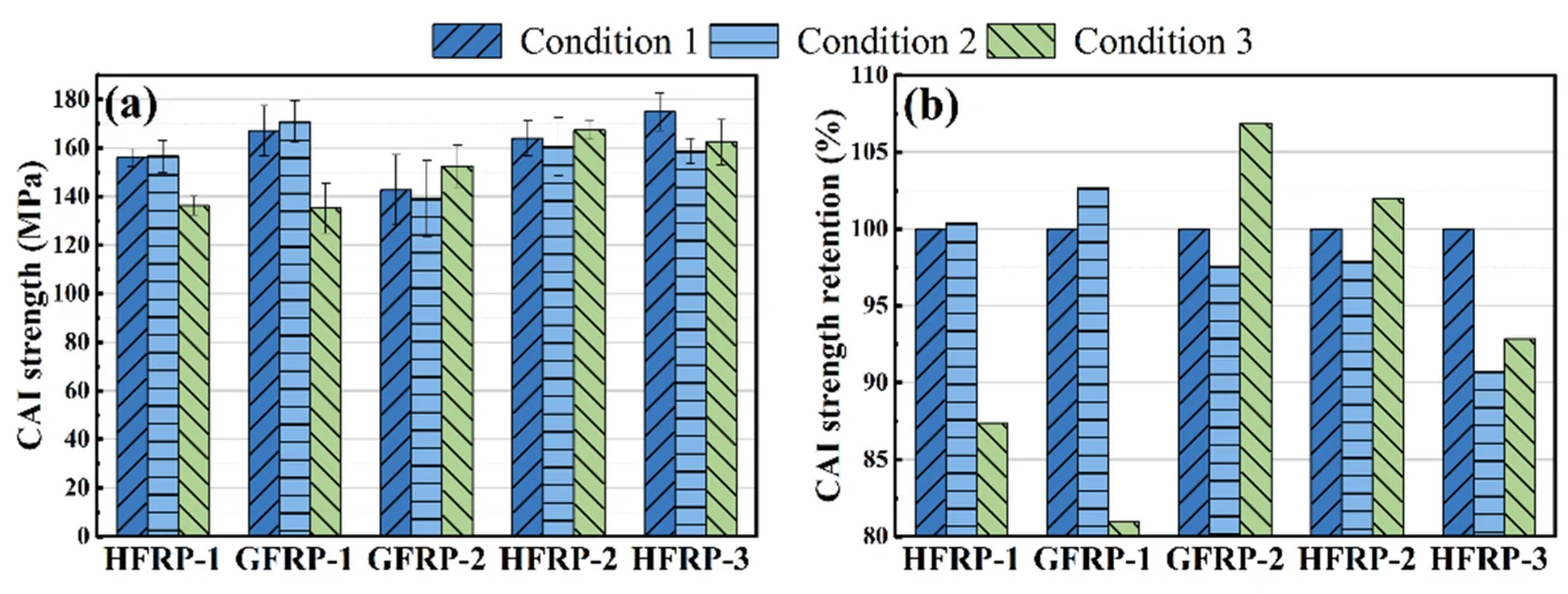
Compression-after-impact properties of GFRP and HFRP laminates under different environmental conditions: (**a**) CAI strength, (**b**) CAI strength retention.

Figure 10a,b presents the CAI strength and corresponding strength retention of the GFRP and HFRP laminates under different environmental conditions. Under Condition 1, the CAI strengths of GFRP-1 and GFRP-2 were 167.08 MPa and 142.67 MPa, respectively, indicating a 17.1% improvement for GFRP-1. The higher CAI strength of GFRP-1 is consistent with its higher compressive strength and ILSS, suggesting better resistance to delamination growth and local instability during post-impact compression. After the introduction of carbon fibers, the CAI strength of HFRP-1 decreased by 6.6% compared with that of GFRP-1, whereas those of HFRP-2 and HFRP-3 increased by 14.9% and 22.6%, respectively, relative to GFRP-2. This trend is generally consistent with the ILSS results discussed previously. For HFRP-1, the incorporation of carbon fibers did not lead to an improvement in interlaminar performance, as reflected by the reductions in both ILSS and CAI compared with GFRP-1. These results suggest that the synergistic effect between CF-1 and GF was relatively limited, with less effective interfacial load transfer and interlaminar constraint. Consequently, impact-induced delamination could propagate more readily during subsequent compression loading, thereby reducing the residual load-carrying capacity of the laminate. After Condition 2, both GFRP laminates exhibited only marginal changes in CAI strength, indicating that the residual post-impact compressive performance was largely retained after moisture preconditioning followed by −50 °C exposure. Similar behavior was observed for HFRP-1 and HFRP-2, whereas the CAI strength of HFRP-3 decreased by 9.2%, suggesting a noticeable reduction in damage tolerance. Because the compressive strength of HFRP-3 remained nearly unchanged after Condition 2, the decrease in its CAI strength is more likely related to the stability of the impact-damaged region during post-impact compression rather than to a loss of intrinsic compressive load-bearing capability. This difference highlights that CAI strength is governed not only by the intrinsic compressive strength of the laminate, but also by the size, morphology, and stability of impact-induced damage. After low-velocity impact, delamination, matrix cracking, and local fiber damage reduce the local bending stiffness of the laminate and create preferential paths for damage growth under compression. A possible post-impact damage evolution process can be inferred from the combined CAI, compressive, ILSS, and SEM results. Low-velocity impact may introduce matrix cracking, interlaminar delamination, and local fiber damage around the impact region, reducing the local bending stiffness of the laminate and providing preferential paths for damage growth. During subsequent compression, these defects may promote delamination extension and local buckling of the damaged sublaminates, leading to premature loss of residual load-bearing capacity.

After Condition 3, the CAI strengths of GFRP-1 and HFRP-1 decreased by 19.1% and 12.6%, respectively, indicating that the VE-1-based laminates were more sensitive to the loss of residual post-impact compressive performance under the designed environmental history. By contrast, the VE-2-based laminates retained higher CAI strengths after Condition 3, suggesting better preservation of residual post-impact compressive performance. The higher CAI retention of the VE-2-based laminates may be related to better matrix/interface integrity and interlaminar constraint, which helped limit delamination growth and local buckling during post-impact compression. This response should still be interpreted as a combined effect of resin behavior, stacking sequence, and impact-damage morphology. The CAI strengths of GFRP-2 and HFRP-2 were 6.9% and 2.0% higher than their Condition 1 values, respectively, while HFRP-3 showed a 7.1% reduction relative to Condition 1 but partially recovered compared with Condition 2.

To facilitate comparison across different tests, the residual mechanical responses of the laminates under Conditions 2 and 3 are summarized in Table 7.

The possible role of moisture uptake was considered qualitatively based on the moisture absorption tendency reported by Zhou et al. [29] for the same laminate systems. Their results showed that the later-stage moisture uptake followed the order HFRP-1 > GFRP-2 > GFRP-1 > HFRP-2 > HFRP-3, indicating that moisture absorption was affected by the resin system, fiber architecture, and carbon/glass interfacial condition. However, this tendency did not show a simple correspondence with the residual mechanical-property retention observed after Condition 3 in the present study. For example, HFRP-2 and HFRP-3 showed relatively low moisture uptake in the previous study, but HFRP-2 still exhibited noticeable reductions in flexural and compressive strength after Condition 3. This suggests that moisture uptake is an important factor, but the residual response is also influenced by stacking configuration, carbon fiber distribution, interface quality, and the dominant failure mode of each test.

### 3.4. Damage Characterization

**Figure 11:**
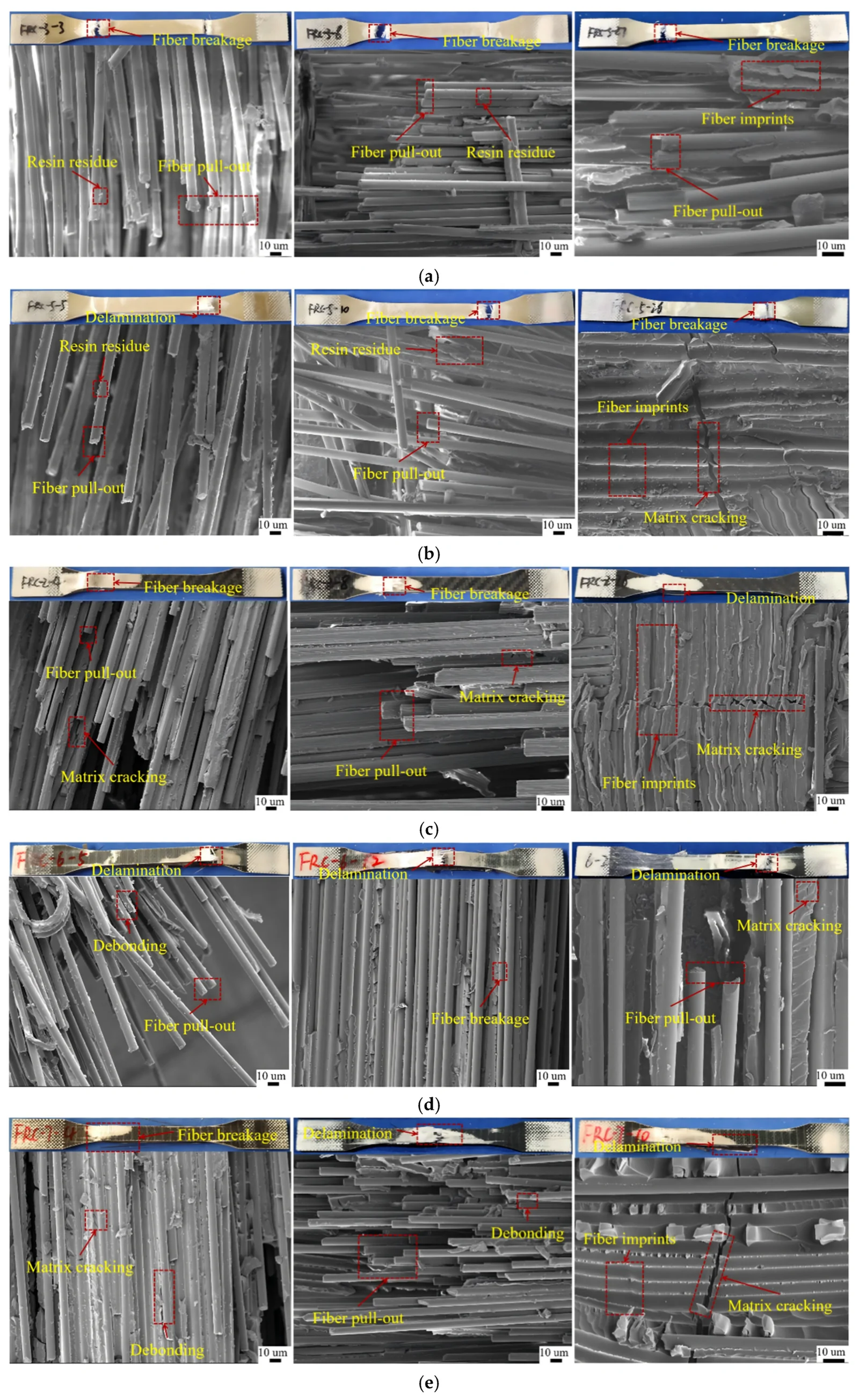
SEM images of tensile fracture surfaces of GFRP and HFRP laminates under different environmental conditions: (**a**) GFRP-1, (**b**) GFRP-2, (**c**) HFRP-1, (**d**) HFRP-2, (**e**) HFRP-3. From left to right, the images correspond to Condition 1, Condition 2, and Condition 3, respectively.

**Figure 12:**
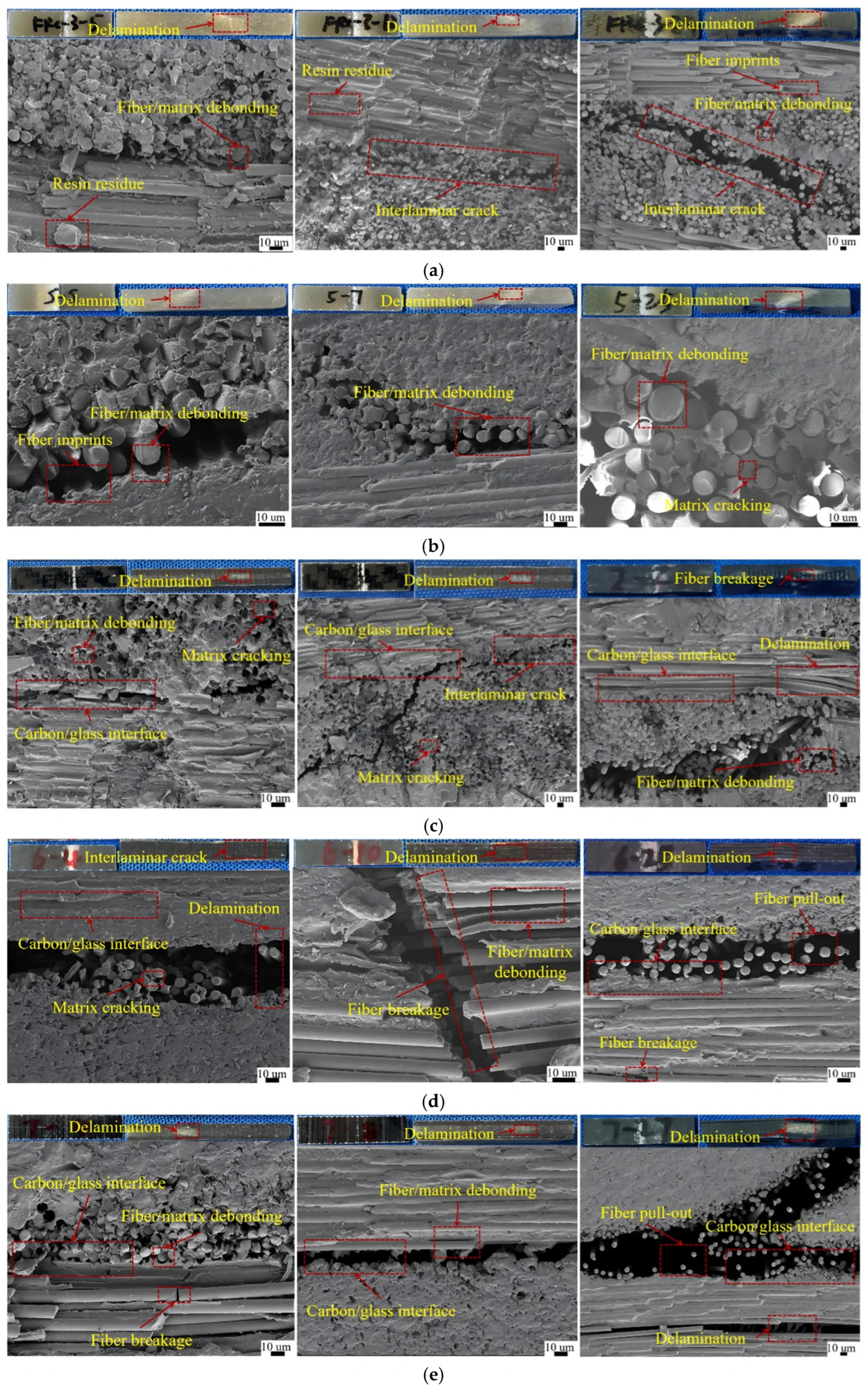
SEM images of interlaminar regions of GFRP and HFRP laminates after ILSS testing under different environmental conditions: (**a**) GFRP-1, (**b**) GFRP-2, (**c**) HFRP-1, (**d**) HFRP-2, (**e**) HFRP-3. From left to right, the images correspond to Condition 1, Condition 2, and Condition 3, respectively.

To clarify the microstructural features related to the changes in mechanical properties of the laminates under different environmental conditions, scanning electron microscopy (SEM) was employed to examine the fracture surfaces of the tensile specimens and the interlaminar regions along the side surfaces of the interlaminar shear specimens. Figure 11 presents the SEM images of the fracture surfaces of the GFRP and HFRP tensile specimens after exposure to Conditions 1, 2, and 3 (from left to right: Condition 1, Condition 2, and Condition 3). The corresponding SEM images of the interlaminar regions in the interlaminar shear specimens are shown in Figure 12 and were used to analyze the fiber-matrix interfacial bonding, interlaminar crack propagation, and environmentally induced microstructural damage evolution.

In some tensile specimens, two rupture sections were observed, which may be associated with the statistical variation in fiber bundle strength and the rapid redistribution of load during tensile failure. Tensile fracture likely initiated from a relatively weak local region, where some fiber bundles or fiber–matrix interfaces first lost their load-bearing capacity. The load carried by this damaged region was then rapidly transferred to the remaining intact fiber bundles and adjacent regions, which could trigger further fiber bundle breakage in another highly stressed section. Therefore, the appearance of two rupture sections reflects a rapid progressive tensile failure process at the fiber-bundle scale. Further examination of the tensile fracture surfaces by SEM revealed that the tensile failure of the GFRP laminates was primarily characterized by fiber fracture, accompanied by localized fiber pull-out and matrix cracking. Under Condition 1, a considerable amount of residual resin remained adhered to the surfaces of the pulled-out fibers, suggesting relatively good interfacial bonding between the glass fibers and the resin matrix. Such interfacial integrity is beneficial for load transfer, allowing the reinforcing fibers to effectively sustain the applied tensile load. After exposure to Condition 2, both GFRP-1 and GFRP-2 still exhibited localized fiber pull-out with a small amount of residual resin attached to the fiber surfaces. No obvious differences in fracture morphology were observed compared with those under Condition 1, indicating that no obvious additional deterioration of the fiber–matrix interface or fiber-dominated tensile failure mechanism was observed after Condition 2. This observation agrees well with the nearly unchanged tensile strengths of the two GFRP laminates after Condition 2 treatment. Following Condition 3, fiber imprints on the matrix, relatively smooth exposed fiber surfaces, and reduced resin residues became more evident, reflecting localized fiber-matrix interfacial debonding. These features suggest that localized interfacial bonding and stress transfer were affected under the Condition 3 environmental history. Nevertheless, because the tensile behavior of the GFRP laminates was still dominated by the continuous glass fibers, the localized interfacial degradation was insufficient to significantly reduce the overall tensile load-bearing capability, which is consistent with the limited variation in tensile strength after Condition 3.

SEM observations of the tensile fracture surfaces of the HFRP laminates revealed that HFRP-1, HFRP-2, and HFRP-3 all exhibited typical tensile failure characteristics under Condition 1, including fiber fracture, localized fiber pull-out, and matrix cracking. These fracture features indicate that tensile loading was primarily sustained by the reinforcing fibers, while effective stress transfer was provided through the fiber-matrix interface. Among the three hybrid laminates, HFRP-2 exhibited more pronounced fiber fracture features, reflecting a superior axial load-bearing capability of the CF-2 hybrid architecture. This observation is consistent with its highest tensile strength and tensile modulus. By comparison, HFRP-1 and HFRP-3 exhibited relatively more fiber pull-out and matrix cracking, suggesting that localized interfacial debonding and crack propagation occurred during tensile failure. After exposure to Condition 2, all HFRP laminates retained fracture morphologies similar to those under Condition 1, with fiber fracture and localized fiber pull-out remaining the dominant failure features. Extensive interfacial debonding and severe matrix cracking were not observed, indicating that no obvious additional change in the tensile failure mechanism was observed after Condition 2. Correspondingly, the tensile strengths of all HFRP laminates remained nearly unchanged after Condition 2 treatment. Following Condition 3, distinct differences in fracture morphology became apparent among the three hybrid laminates. HFRP-1 exhibited more pronounced fiber pull-out and interfacial debonding after Condition 3, implying reduced local fiber–matrix interfacial bonding and less effective stress transfer across the interface. In contrast, HFRP-2 remained dominated by fiber fracture, with only limited localized interfacial debonding, indicating that the carbon fiber plies continued to sustain most of the applied tensile load and that the interfacial damage remained localized. HFRP-3 exhibited relatively more fiber pull-out and interfacial separation, suggesting localized degradation of the fiber-matrix interface after FTC. However, its tensile strength remained stable and even showed a slight increase, indicating that the observed interfacial damage had not become the dominant factor controlling tensile failure. Overall, the fracture morphology observations are in good agreement with the tensile test results, indicating that the tensile response of the hybrid laminates remained primarily governed by the reinforcing fibers, whereas localized fiber–matrix interfacial changes became more evident after Condition 3.

Further examination of the interlaminar regions of the interlaminar shear specimens by SEM revealed that the failure of all laminates was primarily characterized by fiber-matrix interfacial debonding, matrix cracking, interlaminar crack propagation, and localized fiber pull-out, indicating that the interlaminar shear response was mainly governed by the integrity of the fiber-matrix interface and the interlaminar bonding condition. Under Condition 1, a considerable amount of residual resin remained adhered to the fiber surfaces of GFRP-1, and the fiber-matrix interface appeared relatively continuous, indicating relatively good interfacial bonding and efficient interlaminar load transfer. This observation agrees well with the higher ILSS of GFRP-1 compared with GFRP-2. In contrast, GFRP-2 exhibited more exposed fibers and more evident interfacial debonding, reflecting weaker fiber-matrix interfacial bonding and reduced interlaminar load-transfer efficiency. A similar failure mechanism was observed in the HFRP laminates, where matrix cracking, interfacial debonding, and localized interlaminar delamination were frequently initiated near the carbon/glass heterogeneous interfaces. These features suggest that the stiffness mismatch between carbon and glass fibers promoted localized stress concentrations, facilitating crack initiation and propagation along the heterogeneous interfaces. Compared with HFRP-3, HFRP-2 exhibited more pronounced interlaminar delamination, which is more likely associated with the combined effects of its carbon fiber distribution and laminate stacking configuration. Such fracture characteristics are consistent with the lower ILSS of HFRP-2 and further demonstrate that the interlaminar performance of hybrid laminates is governed not only by the carbon fiber content but also by the distribution of the carbon/glass interfaces.

After exposure to Condition 2, only minor changes were observed in the interlaminar fracture morphology of all laminates compared with those under Condition 1. Residual resin remained attached to the fiber surfaces, while no obvious increase in interfacial debonding or interlaminar cracking was detected. These observations indicate that no obvious additional deterioration of the fiber–matrix interface or interlaminar load-transfer path was observed after Condition 2, which agrees well with the nearly unchanged ILSS after Condition 2 treatment. Following Condition 3, more pronounced interfacial debonding, matrix microcracking, and interlaminar crack propagation became evident in all laminates. Relatively smooth exposed fibers and resin peeling marks were also observed in localized regions after Condition 3, reflecting localized deterioration of the fiber–matrix interface after moisture preconditioning followed by FTC. The volumetric expansion of absorbed moisture during freezing may generate localized stresses at the fiber-matrix interfaces and interlaminar regions, thereby promoting the propagation of pre-existing microcracks and interlaminar cracks during repeated freeze–thaw cycling. Overall, the SEM observations agree well with the ILSS results and indicate that the initial damage observed after Condition 3 was mainly associated with interfacial deterioration and damage accumulation under the moisture-preconditioned FTC history.

## 4. Conclusions

In this study, GFRP laminates fabricated with two vinyl ester resin systems and HFRP laminates with three carbon/glass hybrid stacking configurations were investigated to evaluate their early thermomechanical response, residual mechanical performance, and damage evolution under a room-temperature reference condition, 150-day moisture preconditioning followed by −50 °C exposure, and 150-day moisture preconditioning followed by moisture-assisted FTC.

FTIR spectra showed no obvious new absorption bands or systematic peak shifts after environmental conditioning, indicating that no pronounced changes in the main characteristic absorption bands of the resin matrix were detected. DMA results showed that Condition 3, which involved 150-day moisture preconditioning followed by FTC, was associated with varying degrees of reduction in the glass transition temperature ($T_{g}$), with HFRP-1 exhibiting the largest decrease of approximately 5.5 °C. Meanwhile, the FWHM generally increased, with the largest increase of approximately 9.3 °C observed in HFRP-3, suggesting a broader relaxation response and increased thermomechanical heterogeneity after environmental conditioning. Among the investigated laminates, the CF-1/GF hybrid architecture exhibited greater thermomechanical sensitivity to the Condition 3 environmental history. Combined with the FTIR, DMA, and SEM results, the observed property changes were mainly associated with absorbed-moisture-assisted interfacial damage accumulation and local physical damage.

Carbon/glass hybridization significantly improved the strength and stiffness of the laminates, with HFRP-2 exhibiting the highest tensile, compressive, and flexural load-bearing capacities. After Condition 2, most strength properties remained relatively stable, although several indicators, such as the compressive strength of HFRP-2 and the CAI strength of HFRP-3, showed moderate decreases. These results suggest that most laminates retained their residual load-bearing capability after the combined history of 150-day moisture preconditioning and subsequent −50 °C exposure. By comparison, Condition 3 was associated with more apparent changes in matrix-, interface-, and damage-sensitive properties. Tensile and in-plane shear properties remained largely unaffected, whereas compressive, flexural, interlaminar shear, and CAI properties exhibited higher environmental sensitivity. The minimum retention rates of flexural strength and CAI strength were 88.9% and 80.9%, respectively. Compared with the VE-1 system, all VE-2 laminates maintained CAI strength retention rates above 92.9% after Condition 3, indicating better retention of residual post-impact compressive performance under the investigated environmental conditions.

SEM observations showed that fiber–matrix debonding, matrix microcracking, and interlaminar crack propagation became more evident after Condition 3. Together with the ILSS results, these features suggest that matrix/interface-related damage played an important role in the changes of interlaminar performance. The CAI results further indicate that the residual post-impact compressive performance was material-dependent under the designed environmental histories. Overall, the results indicate that the resin system, carbon fiber type, and carbon/glass interface distribution affected the residual mechanical performance and damage response of the laminates under the investigated conditioning histories. These findings provide experimental information for comparing carbon/glass hybrid laminate designs under moisture-assisted low-temperature and FTC conditions. Since deionized water was used as the conditioning medium and the mechanical tests were conducted at approximately 25 °C after environmental conditioning, the present results should be interpreted as the residual mechanical performance and damage response of the laminates after moisture-assisted low-temperature and FTC conditioning. The coupled effects of seawater salinity, dissolved ions and FTC, the in situ load-bearing behavior at −50 °C, and the detailed post-impact damage evolution characterized by delamination area, damage size, load–displacement response, and energy absorption require further investigation.

## Figures and Tables

**Table 1:** Representative studies on FRP composites under low-temperature or FTC-related conditions.

Reference	Material System	Environmental Condition	Tests	Main Findings
Alkhader et al. [15]	CFRP/vinyl ester	Moisture exposure + FTC	Flexural tests	Coupled effects of moisture and FTC
Hasan et al. [18]	GFRP/polyester and vinyl ester	Water preconditioning + FTC	Mechanical; DMA	Greater changes in flexural and compressive properties
Lei et al. [20]	CF/PA6	FTC	Mechanical; dimensional stability; water absorption	Changes in mechanical and moisture-related behavior
Zhou et al. [29]	GFRP and carbon/glass HFRP/vinyl ester	Moisture preconditioning + FTC	Mechanical; FTIR; DMA; SEM	Property retention depended on hybrid stacking
Present study	GFRP and carbon/glass HFRP/two vinyl ester systems	Moisture preconditioning + −50 °C exposure or FTC	Mechanical; FTIR; DMA; SEM; CAI	Resin- and stacking-dependent residual response and damage tolerance

**Table 2:** Basic properties of the vinyl ester resin.

Resin Type	$T_{g}$/°C	Compressive Strength/MPa	Compressive Modulus/MPa	Tensile Strength/MPa	Tensile Modulus/MPa	Coefficient of Thermal Expansion (CTE)/$K^{-1}$
VE-1	123	102–103	3100–3300	65–73	3000–3300	50 × 10^−6^
VE-2	130	102–103	3100–3300	76–82	3100–3300	50 × 10^−6^

**Table 3:** Basic properties of fibers.

Fiber Type	Areal Density ($g/m^{2}$)	Thickness /mm	Tensile Modulus/GPa	Tensile Strength/MPa	Weaving Pattern
CF-1	300	0.21	230	3530	Twill weave
CF-2	400	0.42	230	4900	Non-crimp fabric
GF	280	0.21	53	3000	Satin weave

**Table 4:** The basic parameters of the laminates.

Type	Name	Fiber	Resin	Glass Fiber Volume Fraction (%)	Carbon Fiber Volume Fraction (%)	Lay Up
Carbon/Glass hybrid laminates	HFRP-1	CF-1 and GF	VE-1	16.62	31.47	${\left[G_{1}C_{6}G_{3}\right]}_{s}$
	HFRP-2	CF-2 and GF	VE-2	8.53	41.93	${\left[G_{2}C_{4}\right]}_{s}$
	HFRP-3	CF-2 and GF	VE-2	25.91	22.92	${\left[G_{1}C_{2}G_{5}\right]}_{s}$
Glass fiber laminates	GFRP-1	GF	VE-1	48.98	0	$\left[G_{20}\right]$
	GFRP-2	GF	VE-2	49.22	0	$\left[G_{20}\right]$

**Table 5:** Comparison of representative FTC protocols for FRP composites.

Reference	Composite System	Temperature Range	Number of Cycles	Cycle Duration or Freezing Stage
Alkhader et al. [15]	Carbon fiber/vinyl ester composite	−23 ± 2 °C to room temperature	25, 50, 75 and 100	7–8 h freezing and 4–6 h thawing
Hasan et al. [18]	Vacuum-infused GFRP laminates	−20 °C to 23 °C	100, 200 and 300	13.4 h per cycle
Aniskevich et al. [18,31,32,33,34,35]	Pultruded GFRP profiles	−30 °C to 20 °C	125	24 h per cycle
Present study	GFRP and carbon/glass HFRP laminates	−50 °C to 22 °C	3	72 h freezing and 24 h thawing per cycle

**Table 6:** ${Τ}_{g}$ and Full Width at Half Maximum (FWHM) values of GFRP and HFRP under different environmental conditions.

Type of Specimens	${Τ}_{g}$ and FWHM Under Different Conditions (°C)			
		Condition 1	Condition 2	Condition 3
GFRP-1	${Τ}_{g}$	123.41	122.94	122.59
	FWHM	16.79	19.27	17.97
GFRP-2	${Τ}_{g}$	122.34	119.16	119.41
	FWHM	17.69	20.11	23.37
HFRP-1	${Τ}_{g}$	122.49	121.95	116.95
	FWHM	16.95	21.96	19.76
HFRP-2	${Τ}_{g}$	121.03	121.03	119.86
	FWHM	18.96	24.48	21.28
HFRP-3	${Τ}_{g}$	121.27	121.67	120.94
	FWHM	16.71	20.61	25.96

**Table 7:** Summary of residual mechanical responses of GFRP and HFRP laminates under the designed environmental conditions.

Property	Condition 2	Condition 3	Main Implication
Tensile	No marked change	No marked change	Low sensitivity
Compression	HFRP-2 decreased	Different trends among laminates	Material-dependent response
Flexural	No marked change	HFRP-2 decreased	Sensitive to FTC
In-plane shear	Strength and modulus increased	Close to Condition 2	Good retention
ILSS	No marked change	Slight decrease	Interface-related sensitivity
CAI	HFRP-3 decreased	Larger loss in VE-1 laminates	Damage-tolerance sensitivity

## Data Availability

The data that support the findings of this study are available from the corresponding author upon reasonable request.
